# Multimodal Locomotion and Active Targeted Thermal Control of Magnetic Agents for Biomedical Applications

**DOI:** 10.1002/advs.202103863

**Published:** 2022-01-20

**Authors:** Armando Ramos‐Sebastian, So‐Jung Gwak, Sung Hoon Kim

**Affiliations:** ^1^ Department of Electronics Convergence Engineering Wonkwang University Iksan 54538 Republic of Korea; ^2^ Department of Chemical Engineering Wonkwang University Iksan 54538 Republic of Korea; ^3^ Wonkwang Institute of Materials Science and Technology Wonkwang University Iksan 54538 Republic of Korea; ^4^ Present address: Department of Convergence Technology Engineering Jeonbuk National University Jeonju 54896 Republic of Korea

**Keywords:** electromagnetic system, magnetic hyperthermia, magnetic microrobots, magnetic nanoparticles

## Abstract

Magnetic microrobots can be miniaturized to a nanometric scale owing to their wireless actuation, thereby rendering them ideal for numerous biomedical applications. As a result, nowadays, there exist several mechano‐electromagnetic systems for their actuation. However, magnetic actuation is not sufficient for implementation in biomedical applications, and further functionalities such as imaging and heating are required. This study proposes a multimodal electromagnetic system comprised of three pairs of Helmholtz coils, a pair of Maxwell coils, and a high‐frequency solenoid to realize multimodal locomotion and heating control of magnetic microrobots. The system produces different configurations of magnetic fields that can generate magnetic forces and torques for the multimodal locomotion of magnetic microrobots, as well as generate magnetic traps that can control the locomotion of magnetic swarms. Furthermore, these magnetic fields are employed to control the magnetization of magnetic nanoparticles, affecting their magnetic relaxation mechanisms and diminishing their thermal properties. Thus, the system enables the control of the temperature increase of soft‐magnetic materials and selective heating of magnetic microrobots at different positions, while suppressing the heating properties of magnetic nanoparticles located at undesired areas.

## Graphical abstract

The multimodal electromagnetic system (MECS) is a system comprised of three pairs of Helmholtz coils, a pair of Maxwell coils, and a high‐frequency solenoid. The system produces different configurations of magnetic fields that can generate magnetic forces, magnetic torques, and heat release for the multimodal locomotion and heating control of magnetic agents for biomedical applications.

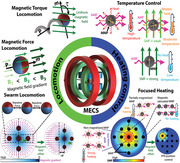

## 1 Introduction

Several types of untethered microrobots such as biological (cell‐driven), optical, acoustic, and magnetic‐driven magnetic microrobots have been developed and researched extensively for biomedical applications. Although such types of magnetic microrobots exhibit great performance in vitro applications, most are not suitable for in vivo applications. Optical microrobots are limited to applications that do not require high tissue penetration depths^[^ ^1^, ^2^ ^]^ owing to the potential harm to the superficial tissue; while acoustic actuation has limited transmission through hard tissues.^[^ ^3^ ^]^ Furthermore, strong ultrasound can cause heating and tissue damage at the focal point.^[^ ^4^ ^]^ Moreover, biological microrobots cannot be actively controlled and their actuation force is weaker than the aforementioned methods; thus, they are typically combined with synthetical elements to improve their controllability and force.^[^ ^5^, ^6^, ^7^ ^]^

Magnetic fields can penetrate within any region in the human body, and the implementation of a strong magnetic field, up to several Tesla, does not pose any considerable adverse effects.^[^ ^8^, ^9^ ^]^ Consequently, magnetically driven microrobots can safely achieve higher forces and exhibit a faster motion compared to optical or acoustically driven microrobots.^[^ ^1^ ^]^ Thus, many researchers rely on magnetic‐driven locomotion for microrobots, while combining it with other physical and chemical mechanisms to enable in vivo tracking (e.g., fluorescent, X‐ray, and acoustic imaging methods)^[^ ^10^, ^11^, ^12^ ^]^ and drug delivery applications (e.g., acoustic, chemical, and photothermal based drug release).^[^ ^13^, ^14^, ^15^ ^]^ Consequently, several configurations of electromagnetic actuation systems have been developed for the control of magnetic microrobots.

An electromagnetic actuation system composed of permanent magnets can easily achieve high magnetic field values; however, the produced magnetic fields cannot be suppressed, and their magnetic field intensity must be controlled through mechanical mechanisms.^[^ ^16^, ^17^ ^]^ Further, researchers have also developed electromagnetic actuation systems composed of several distributed solenoidal coils with ferromagnetic cores directed toward the working space that can create moderately high magnetic fields (hundreds of mT). However, because of their voluminous size, they can be employed under small working scales only, and thus are used for mostly in vitro applications or in vivo surgery in small superficial zones of the body, such as the eye,^[^ ^18^, ^19^ ^]^ or partial sections of the body.^[^ ^20^, ^21^ ^]^ Another configuration of coils extensively used is based on spatial configurations of pairs of electromagnets, such as Helmholtz, Maxwell, and saddle coils. Maxwell coils create uniform magnetic field gradients, whereas Helmholtz and saddle coils produce uniform magnetic fields and nonuniform magnetic field gradients. Although the magnetic field these coils produce is smaller than distributed ferromagnetic core electromagnet systems, researchers prefer to use these types of coils owing to ease of control and their capacity to handle a larger working space.^[^ ^22^, ^23^ ^]^

The development of new magnetic‐based medical technologies such as magnetic particle imaging^[^ ^24^, ^25^ ^]^ and magnetic hyperthermia,^[^ ^26^, ^27^ ^]^ have reduced the reliance of magnetic microrobots on different types of physical or chemical phenomena for their biomedical applications. Hence, biomedical magnetic microrobots can be controlled exclusively by magnetic fields, reducing the equipment requirements because a single device that is capable of performing all the necessary tasks, or most of them, can be manufactured. Thus, recently, researchers are working on developing multifunctional electromagnetic systems capable not only of magnetic locomotion control for magnetic microrobots but also the ability to track their position when inside the human body.^[^ ^28^, ^29^ ^]^

Magnetic microrobots designed for magnetic hyperthermia applications comprise of magnetic nanoparticles (MNPs).^[^ ^30^, ^31^ ^]^ However, even when functionalized with biological markers, many of the MNPs drift to healthy parts of the body, mainly the liver and spleen.^[^ ^32^, ^33^ ^]^ Thus, to avoid damaging the tissue, suppressing the heating in such regions caused by the exposure to a high‐frequency alternating magnetic field (AMF) is crucial. It has been demonstrated that partial magnetization of MNP alters its thermal properties, either by enhancing or diminishing them. The application of a bias DC static magnetic field (SMF) can induce different types of assemblies in the MNP and align their easy axis in the direction of the AMF, improving their heating performance.^[^ ^34^, ^35^, ^36^ ^]^ However, the constant application of an SMF also decreases the heat released by the MNP, reaching negligible values when the MNP are magnetically saturated.^[^ ^37^, ^38^, ^39^ ^]^ Therefore, to achieve selective heating of MNP, certain researchers have emulated a principle of magnetic particle imaging: the use of a field‐free region (FFR). An FFR is a special magnetic field distribution wherein the magnetic field is zero at its center and increases in all directions, such that MNP outside of the FFR are magnetically saturated, and hence heat can be selectively applied only to MNP within the FFR^[^ ^40^, ^41^ ^]^ (see Section S1 in the Supporting Information). However, previous reported research has realized selective heating of MNP by using permanent magnets and mechanical mechanisms to control the position of the FFR, without the ability to control the size of the FFR or to carry out locomotion control of magnetic microrobots.

To the best of our knowledge, no electromagnetic actuation system capable of controlling the heating properties and the magnetic locomotion of magnetic microrobots (both in the 3D space) has been developed. This is primarily because of the high magnetic field gradient distribution that is required for the generation of an FFR, typically above 2 mT mm^−1^, and the undesired heating due to eddy currents that the AMF can induce in the neighboring coils.^[^ ^42^, ^43^ ^]^ Consequently, the FFR is usually produced using neodymium magnets^[^ ^44^, ^45^ ^]^ or ferromagnetic core electromagnets,^[^ ^29^, ^46^ ^]^ such that they can be placed sufficiently far from the AMF generating coil while still producing a high magnetic field gradient. However, the use of magnets and ferromagnetic core electromagnets results in the reduction of the working space and accessibility for the operator and blocks visibility from the working area, while also making it harder to add more coils into the system for additional functionality.

**Figure 1:**
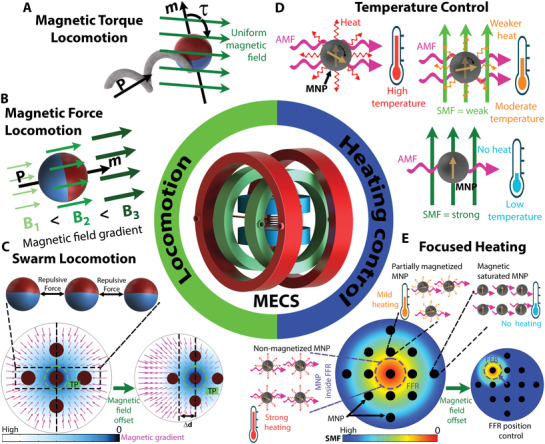
Multimodal electromagnetic system (MECS). A) System generates uniform magnetic fields to control the locomotion of magnetic robots through magnetic torque *τ*. B) System generates magnetic field gradients to control the locomotion of magnetic robots through a magnetic force. C) System generates a magnetic field gradient distribution that pushes magnetized robots toward a “trapping point” (TP), whose position can be controlled by superpositioning a uniform magnetic field. D) The system generates high‐frequency alternating magnetic fields (AMF) and static magnetic fields (SMF) to control the heat produced by MNP. E) System generates a field‐free region (FFR), which magnetically saturates MNP outside of the FFR, focusing heating to MNP within the FFR.

Because of the varying environments under which the microrobots are required to operate, magnetic microrobots need to adapt their locomotion mechanism accordingly; thus, the development of magnetic systems that allow their multimodal locomotion is an increasing necessity.^[^ ^47^ ^]^ In this study, we propose a novel multimodal electromagnetic system (MECS) for the multimodal locomotion of single and swarm magnetic microrobots, as well as the control of the heating properties of soft‐magnetic microrobots and magnetic nanoparticles, as shown in **Figure** **1**. MECS comprises of a high‐frequency solenoid coil, three pairs of Helmholtz coils, and a pair of Maxwell coils. Each coil in the three pairs of Helmholtz coils is controlled independently, and thereby several configurations of uniform magnetic fields and magnetic field gradient distributions can be generated for the multimodal locomotion of magnetic microrobots. For example, using only uniform fields, a microrobot can be controlled exclusively via magnetic torque (Figure 1A), whereas through the generation of magnetic field gradients, a microrobot can be actuated via magnetic forces (Figure 1B). Furthermore, the combination of these fields can generate magnetic trapping points (TP) than can also produce the self‐assembly and control the locomotion of swarms of magnetic microrobots, as shown in Figure 1C, which has not been previously reported in static coil systems.^[^ ^48^ ^]^

A solenoid coil generates a high‐frequency alternating magnetic field (AMF) that induces heating of the MNP. Further, the heat released by the MNPs is controlled via an applied time constant uniform field, called a static magnetic field (SMF) which magnetizes the MNPs and thereby hinders their heating properties. As shown in Figure 1D, with an increase in the SMF field, the heat released by the MNP decreases, until they become magnetically saturated, and the released heat becomes zero. The pair of Maxwell coils are used to generate an FFR to focus the heating of MNP to the center of the working space, and by superpositioning a 3D SMF, the location of the FFR can be shifted to target any desired region within the working space (Figure 1E). This is the first system reported for the simultaneous 3D multimodal locomotion of magnetic microrobots and the control of their thermal properties, which makes it suitable for biomedical applications like targeted drug release, clog removal, and selective magnetic hyperthermia applications. Particularly, in the case of magnetic hyperthermia, the system can provide the localized thermal stimulus while avoiding damaging healthy cells. Furthermore, it is the first reported coreless electromagnet configuration for the generation of an FFR.

## 2 Constitution of MECS

**Figure 2:**
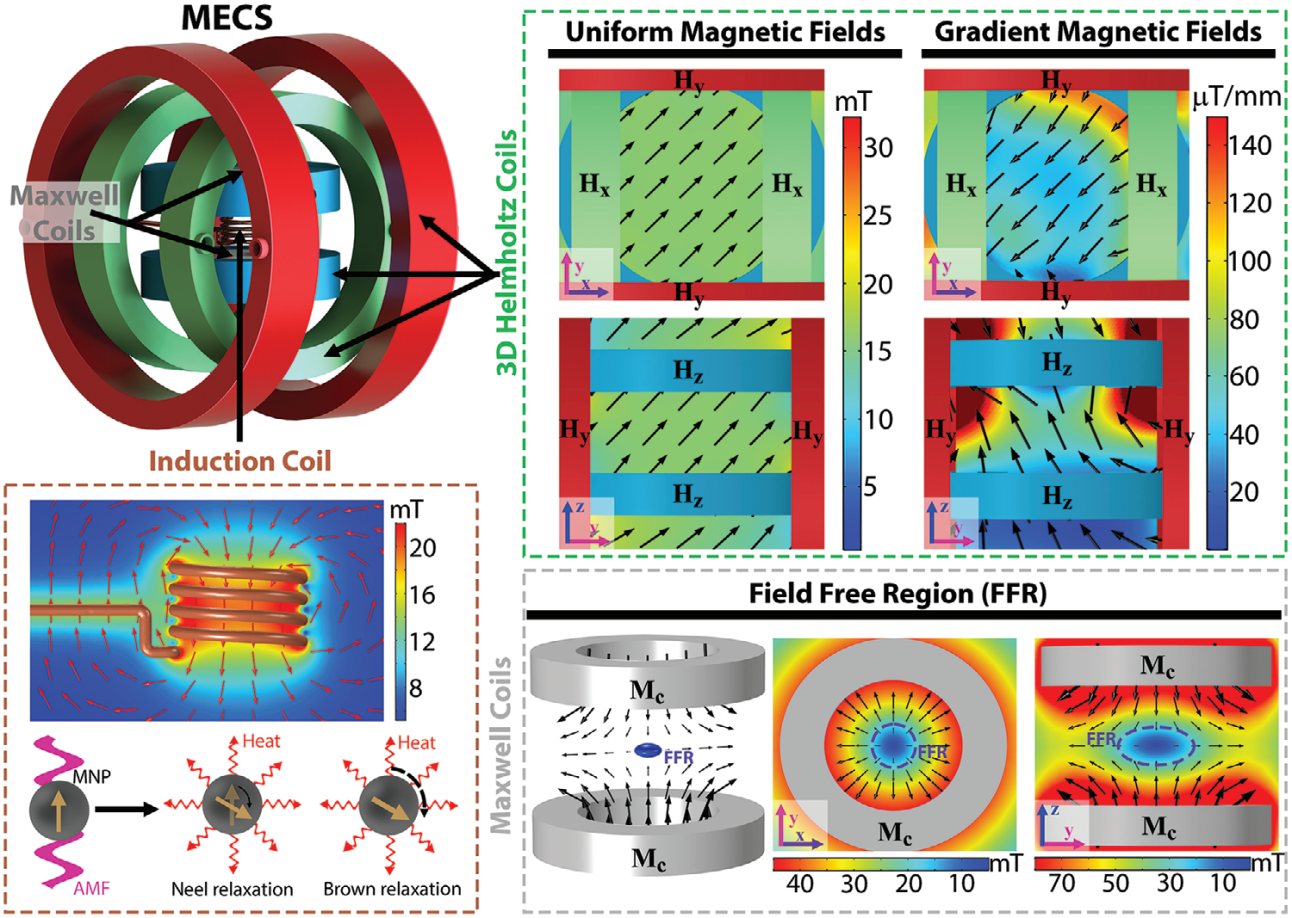
Composition of MECS. MECS comprises an induction coil for the heating of magnetic materials, three pairs of orthogonal Helmholtz coils for the production of uniform and magnetic field gradients, and a pair of Maxwell coils for the generation of a field‐free region (FFR).

We developed a system to achieve multimodal locomotion of magnetic microrobots and control of their thermal properties, as depicted in **Figure** **2**. The induction coil is part of a commercial system (Osung Hitech, OSH‐R5) consisting of a solenoid coil capable of producing high‐frequency alternating magnetic fields (AMF) up to 22 kA m^−1^ at a fixed frequency of 200 kHz, within a cylindrical working space with radius of 60 mm and height of 50 mm. The AMF is responsible for the Néel relaxation (rotation of the magnetic moment) and Brown relaxation (physical rotation) of magnetic nanoparticles (MNP), the primary mechanisms through which monodomain MNP produce heat. In addition, the AMF can produce heating of ferromagnetic materials via hysteresis loss and the heating of electric conductive materials through eddy current mechanisms.

The pair of Maxwell coils are positioned with their axis matching the *Z*‐axis. They create a point with zero magnetic field at its center and a linearly increasing magnetic field in all directions, thereby producing a constant magnetic gradient distribution. The coils have a mean radius of 70 mm and 200 turns and are capable of producing a maximum magnetic field gradient *G* = [1.5 1.5 3] mT mm^−1^ for a working space of 30 mm × 30 mm × 30 mm. Further, at the center of the axis of the coil, there exists a region where the magnetic field is too low to magnetically saturate MNP (circular for the *XY* plane and ellipsoidal for *ZY* or *ZX* plane), which is known as the field‐free region (FFR). Moreover, the area and volume of the FFR depend on the properties of the used MNP.

A pair of Helmholtz coils are employed for each axis of the 3D space, namely, Hx, Hy, and Hz, with their axis located at the *X*‐, *Y*‐, and *Z*‐axes, respectively. Each pair of coils was designed with the ability to move the FFR through the working space, and thus, to move the center of FFR along the desired working space, the coils had to produce a maximum magnetic field *B* = [45 45 90] mT. Consequently, the pair of coils Hx, Hy, and Hz were designed with a mean radius of 14, 22, and 30 cm, and a number of turns of 583, 430, and 602, respectively. Because each coil in the system could be controlled individually, in addition to the magnetic torque control commonly used in 3D Helmholtz system, the pair of Helmholtz coils produced a nonuniform magnetic field gradient distribution, which was used to implement the magnetic force control of the magnetic robots as well. Thus, the system achieved 6 degrees of freedom for the locomotion of magnetic robots. For a more detailed description and images of the system, refer to Section S2 in the Supporting Information.

## 3 Locomotion of Magnetic Millirobots

### 3.1 Single Robot Multimodal Locomotion

In this study, the experiments were performed using magnetic millirobots; however, the same control mechanisms apply to magnetic microrobots as well. First, we prove the ability of our system to control magnetic millirobots considering the two most used magnetic locomotion mechanisms: magnetic torque locomotion using a helicoidal millirobot (MR1), and magnetic force locomotion using a spherical magnetic millirobot (MR2) (see material and methods and Section 3 in the Supporting Information). For the closed‐loop locomotion control of a helicoidal magnetic millirobot in a boundless liquid, we use the following equation

(1) $$F_{m}=\left[\begin{array}{c} \lambda \omega \sin\left(\psi \right)\\ \lambda \omega \cos\left(\psi \right) \end{array}\right]$$

where *λ* is a constant that depends on the geometric properties of the robot and the properties of the fluid, *Ψ* is the rotation axis of the rotating magnetic field (RMF), and the angular frequency *ω* is

(2) $$\omega =\sqrt{{\left(\left(\omega_{0}+\omega_{l}\right)\sin\left(\psi \right)\right)}^{2}+{\left(\left(\omega_{0}+\omega_{l}\right)\cos\left(\psi \right)\right)}^{2}}$$

where *ω*_0_ is the gravity compensation angular frequency of the robot and *ω*_l_ is the locomotion angular frequency.

For the closed‐loop magnetic force locomotion of the spherical millirobot in a boundless liquid, we used the pair of coils Hz to generate a magnetic force in the 3D space, and subsequently, by controlling the orientation of the millirobot, we controlled its propulsion direction. For this, we calculate the magnetic force using the following equation

(3) $$F_{m}=m\cdot G_{m}=m\cdot {\left[\begin{array}{cc} g_{lr} & g_{0}+g_{lz} \end{array}\right]}^{T}$$

where **m** is the magnetic moment of the millirobot, *g*_0_ is the gravity compensation magnetic gradient, and *g*_l_ is the locomotive magnetic gradient. For the detailed procedure regarding the deduction of the equations and a more comprehensive model along with the discussion of wall effects and interaction between robots, refer to Section S4 in the Supporting Information.

**Figure 3:**
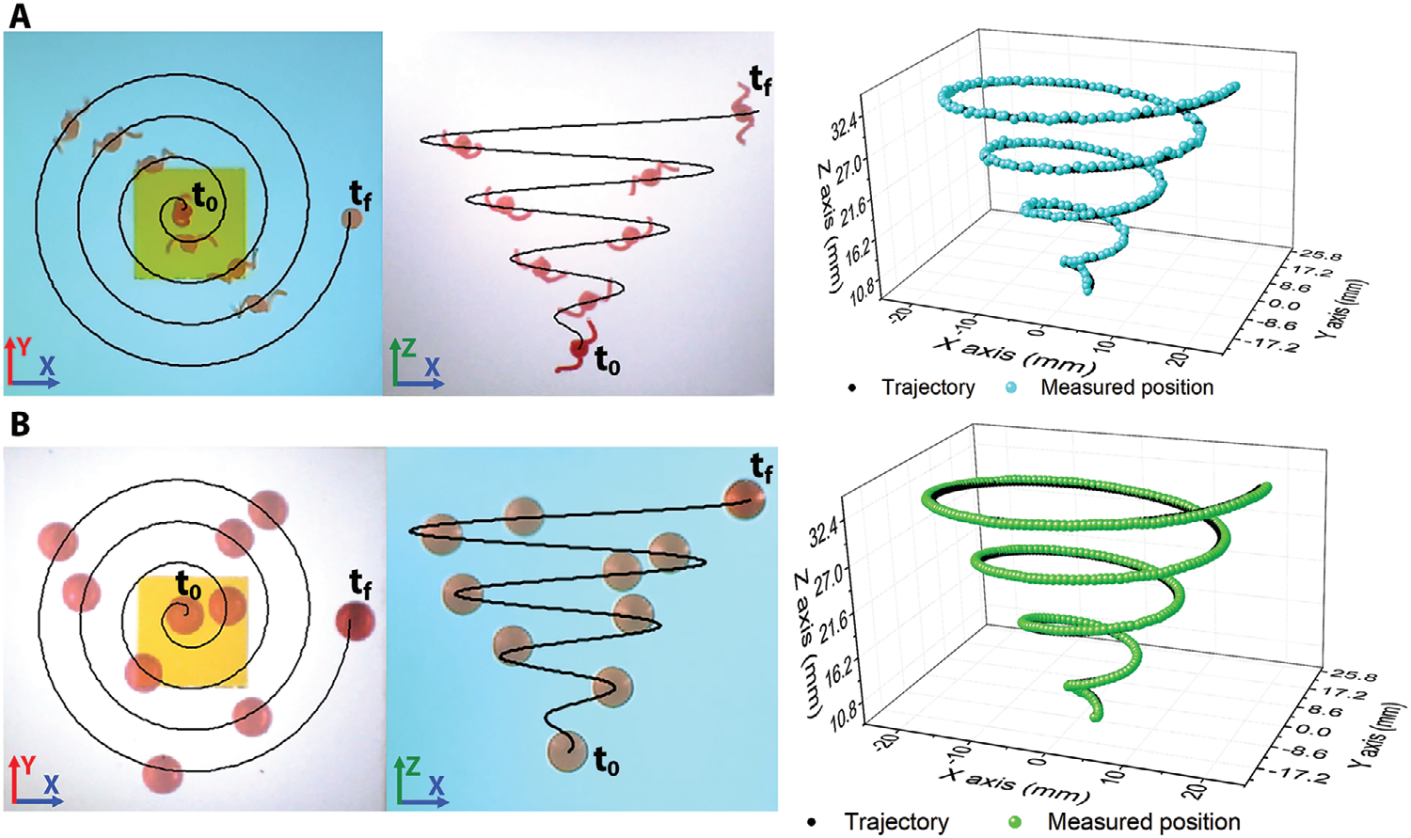
Magnetic torque and magnetic force locomotion. Timelapse images and 3D graph of the magnetic force A) and magnetic torque B) locomotion of a spherical magnetic millirobot.

We designed a 3D spiral trajectory and tested the locomotion of MR1 and MR2 inside silicone oil with a kinematic viscosity (*ν*) of 2000 mm^2^ s^−1^ using a PID controller, as shown in **Figure** **3** (see video S1 in the Supporting Information). Further, we performed each experiment five times and measured the position of the millirobots with respect the desired trajectory. Thereafter, based on the measured data, we calculated the mean error between the measured position and the input trajectory, as well as its locomotion speed. Figure 3A shows the timelapse images of MR1 in the *YX* and *ZX* planes and a graph depicting the measured position while the robot follows the designed trajectory, whereas Figure 3B shows the corresponding images for the locomotion of MR2. The results indicate that MR1 moved with an average error of 310 µm, at a mean speed of 3.76 mm s^−1^, whereas MR2 showed a mean error of 580 µm and a mean speed of 7.7 mm s^−1^.

### 3.2 Magnetic Swarm Locomotion: Magnetic Trapping Point

**Figure 4:**
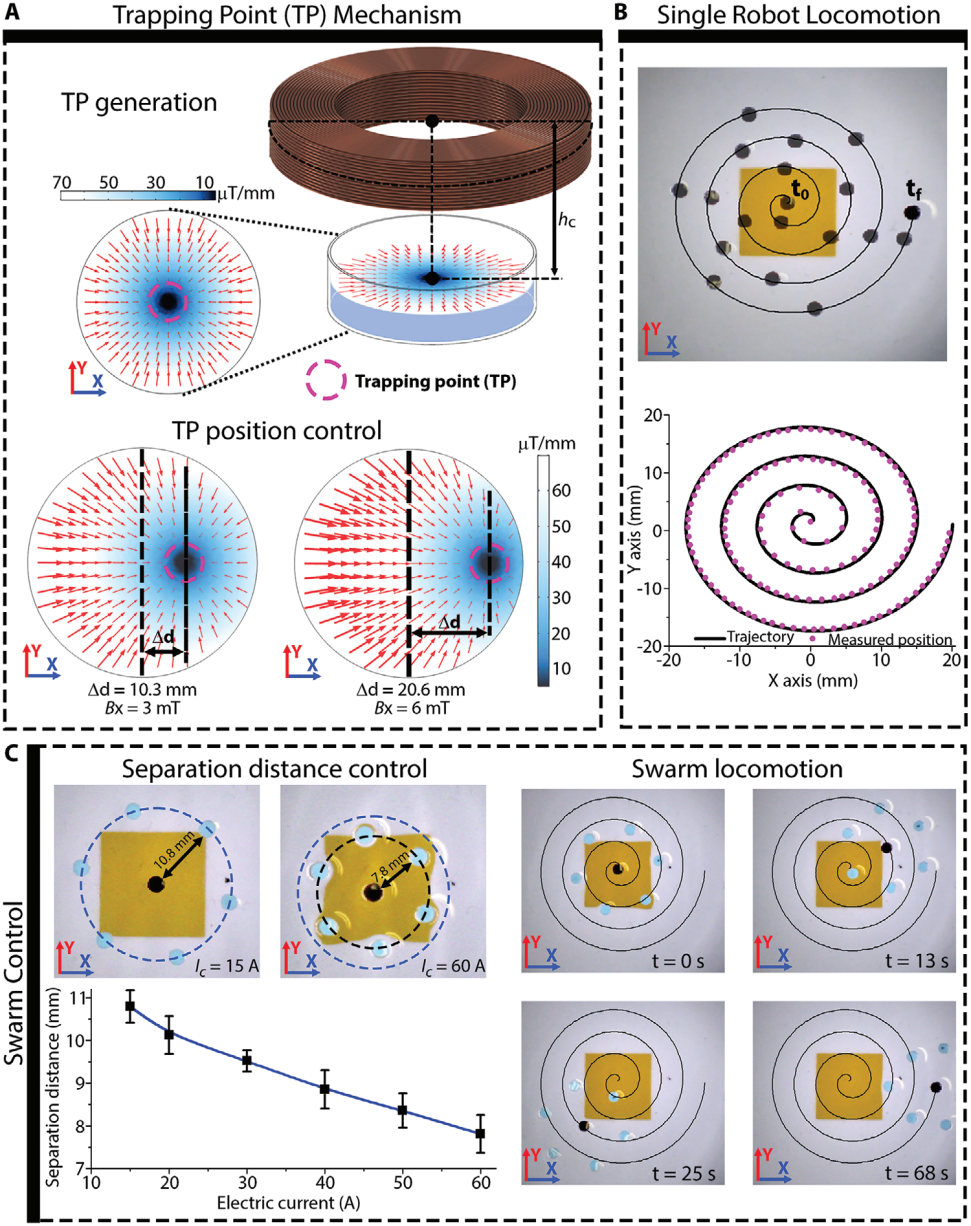
Magnetic trapping point locomotion. A) Trapping point (TP) mechanism: generation of TP and position control. B) Locomotion of a single millirobot using the TP. C) Control of the separation distance between magnetic millirobots in a swarm and locomotion using TP.

We tested the 2D swarm locomotion of magnetic millirobots by developing a magnetic TP, as described in **Figure** **4**, for the locomotion of millirobots at the surface of a liquid (see video S2 in the Supporting Information). The upper maxwell coil (Mc_1_) was used to generate a magnetic field gradient distribution that caused any magnetized object to move toward the axis of the coil, and thereby, a magnetic TP. Further, a uniform field created by the Helmholtz coils was added to control the position of the TP and the position of a magnetic millirobot. Figure 4A shows the magnetic gradient distribution generated by Mc_1_ when a current of 40 A flows through the coil. Subsequently, on applying a magnetic field of 3 and 6 mT in the *X* direction, the TP moved 10.3 and 20.6 mm, respectively, in the *X* direction. Thus, employing this proposed mechanism, we controlled the locomotion of a magnetic millirobot (MR3) along a 2D spiral trajectory, at the surface of silicone oil with a kinematic viscosity of 5 mm^2^ s^−1^. Figure 4B shows the timelapse images of the locomotion of MR3, and the measured position along the spiral trajectory. Thereafter, on repeating the experiment five times, we measured an average error mean speed of 402 µm and 3.27 mm s^−1^, respectively.

If many identical millirobots (MR3 in our case) are placed in the working space of the TP, the magnetic field gradient causes the robots to move toward the TP, while the millirobots repel each other and self‐assemble with the center of the swarm located at the TP. The self‐assembled swarm comprises a lead magnet located at the TP with the remaining millirobots orbiting around the lead magnet. By controlling the position of the TP, the locomotion of the magnetic swarm (MS1) can be controlled, as shown in Figure 4C. Further, on increasing the magnitude of the electric current flowing through Mc_1_ the magnitude of the magnetic gradient is increased, which further increases the magnetic force that causes the millirobots to move toward the TP and decreases the separation distance between the lead magnet (black color) and the orbiting millirobots (blue color). As shown in Figure 4C, when the current flowing in the coil is 15 A, the mean separation distance between the magnets is 10.8 mm, whereas for a current of 60 A, the separation distance decreases to 7.8 mm.

Figure 4C also shows the timelapse images of MS1 as it moves along the designed spiral trajectory. We tested the locomotion of MS1 along the same spiral trajectory used for MS1 in the same silicone oil. After performing the experiment five times, we measured a mean error of 433 µm and a mean speed of 3.66 mm s^−1^. These values were similar to those of MR3, which was expected, as the millirobots that conform MS1 are all identical to MR3.

## 4 Heating Control of MNP and Magnetic Millirobots

### 4.1 Temperature Control of Magnetic Nanoparticles Using an SMF

**Figure 5:**
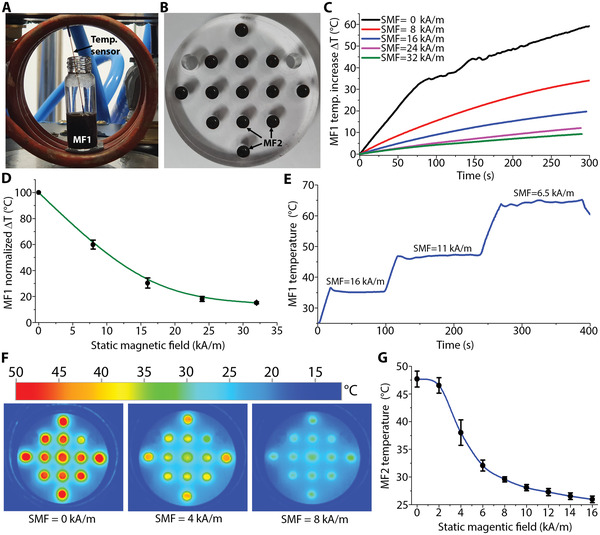
Temperature control of MNP using an SMF. A) Vial containing a magnetic fluid (MF1) inside the induction coil. B) Acrylic container with holes containing a superparamagnetic magnetic fluid (MF2). C) Temperature increase of MF1 when exposed to an AMF for different values of SMF. D) Normalized temperature increase with respect the applied SMF for MF1. E) Temperature control of MF1 using SMF. F) Thermal images of MF2 when exposed to an AMF for different values of SMF. G) Measured temperature for MF2 when exposed to an AMF for different values of SMF.

Two different magnetic fluids were used to test the ability of our system to control the heating produced by MNP when exposed to an AMF, by controlling their magnetization. The magnetic fluid 1 (MF1) is a water‐based ferromagnetic fluid with 20% weight concentration of noncoated MNPs with a core size in the range of 15–20 nm (US Research Nanomaterials, Inc., USA) (**Figure** **5**A). Because the MNP of MF1 are not coated, the MNPs interact with each other in the fluid, which increases the required SMF value for achieving magnetic saturation. Therefore, we also performed experiments on a second magnetic fluid (MF2), which is a decane‐based superparamagnetic fluid with oleic acid‐coated 25 nm spherical MNPs (Ocean Nano Tech, USA) and an iron concentration of 50 mg mL^−1^ (see the Experimental Section). We put 2 mL of MF1 inside a 4 mL vial and used an optical fiber temperature sensor FTX‐300‐LUX+ (OSENSA Innovations Corp, Canada) to measure its temperature as it increased, when exposed to an AMF, as shown in Figure 5A. For experiments with MF2, 13 holes with a diameter of 5 mm were made inside an acrylic container, 120 µL of MF2 was placed in each hole, as shown in Figure 5B.

To obtain reliable data, five samples of MF1 were prepared and the same experiments were performed for each sample. Figure 5C shows the temperature increase (∆*T*) of one sample of MF1 when exposed to an AMF of 16 kA m^−1^ for different values of SMF during a period of 300 s. As observed, in the absence of SMF (SMF = 0), ∆*T* of MF1 reached 59.48 °C, whereas it decreased to 34.12, 19.71, 12.09, and 9.34 °C for applied SMF values of 0, 8, 16, 24, and 32 kA m^−1^, respectively. Because the MNPs in MF1 were not individually coated, their distribution in the fluid was not uniform and the temperature reached by each vial of MF1 was different (see Section 5 in the Supporting Information). Thus, we normalized the measured ∆*T* of each vial and consequently obtained the graph shown on Figure 5D, which shows that the decrease in ∆*T* with respect to the applied values of SMF was proportionally the same for all the vials. Thereafter, an additional experiment was performed to demonstrate that the temperature of MNPs can be dynamically controlled via the application of an SMF, as shown in Figure 5E (see video S3 in the Supporting Information). In particular, high‐precision temperature control of MNPs has not been reported elsewhere for magnetic hyperthermia. First, the SMF was set to 0 kA m^−1^, allowing MF1 to increase its temperature above 36 °C, following which the SMF was changed to 16 kA m^−1^ and the temperature settled at ≈35 °C. Further, applying the same procedure, the temperature increased to and settled at ≈46 and 64 °C for SMFs of 11 and 6.5 kA m^−1^, respectively.

To evaluate the performance of MF2, we used an AMF of 10.88 kA m^−1^, and measured the temperature attained at every hole of the acryl container filled with MF2 for various values of SMF. Figure 5F shows the thermal pictures of MF2 for SMF of 0, 4, and 8 kA m^−1^, whereas Figure 5G shows the mean value of the measured temperature at each hole containing MF2, for SMFs ranging from 0 to 16 kA m^−1^. For an SMF value of zero, all the points show a red color in the thermal picture and their average temperature was 47.7 °C. In contrast, for an SMF of 4 kA m^−1^, the color turns to yellow for the nine points at the center of the container, and orange for the four points located closest to the edge of the container and the induction coil (where the AMF is higher), reaching an average temperature of 38 °C. Furthermore, for SMF of 8 kA m^−1^, the points are light green, having a mean temperature of 29.5 °C. For the individual temperature at each point, refer to Section S5 in the Supporting Information.

### 4.2 Focused Heating of MNP Using FFR Control

**Figure 6:**
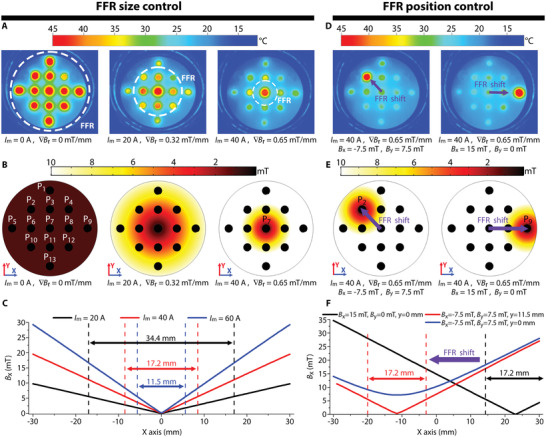
Focused heating of MNP. Thermal images A), simulated FFR B), and magnetic field distribution along the *X*‐axis C) when applying an FFR with different magnetic field gradient ∇*B_r_* values. Thermal images D), simulated FFR E), and magnetic field distribution along the *X*‐axis F) when shifting the position of the FFR for ∇*B_r_* = 0.65 mT mm^−1^.

After analyzing the results from the previous section for the control of the thermal properties of MF2, we observed that for an SMF of 8 kA m^−1^ or higher, the average temperature of MF2 was below 30 °C. Thus, we set it as the “turn‐off” magnetic field for the thermal properties of MF2. Subsequently, we performed simulations of the magnetic field distribution created by the pair of Maxwell coils when different values of electric current flowed through them, to estimate the radius and position of the FFR in the *XY* plane. Thereafter, we performed experiments to verify the size and position of the FFR for MF2 when exposed to an AMF of 10.88 kA m^−1^, as described in **Figure** **6** (see video S4 in the Supporting Information). The experiments were performed using the same acrylic container, and each point was enumerated to facilitate their identification, starting from the upper point (P_1_), from the left to right, till the last point located at the lowest position (P_13_).

As the magnetic gradient is the same in the *X* and *Y* directions, the magnetic field only changes with the radial distance *r* ($r=\sqrt{x^{2}+y^{2}}$) from the center of the FFR; thus, we can define the field gradient distribution in the *XY* plane by using the magnetic field gradient of *r*, ∇*B_r_*. Figure 6A shows thermal pictures obtained after exposing the container with MF2 to the AMF for a period of 120 s, while applying an FFR with a ∇*B_r_* of 0, 0.32, and 0.65 mT mm^−1^, generated when the current flowing in the pair of maxwell coils (*I*_m_) was 0, 20, and 40 A, respectively.

Figure 6B shows the simulated magnetic field distribution, predicting the heated areas in a range of colors from burgundy (maximum heating) to white (no heating). Using the data from the simulations, we calculated the FFR with a diameter of 34.4, 17.2, and 11.5 mm for ∇*B_r_* values of 0, 0.32, and 0.65 mT mm^−1^, respectively, as shown in Figure 6C. The simulation images exhibit great similarity to the thermal images. For ∇*B_r_* of zero, all the points containing MF2 are heated to a mean temperature of 47.6 °C, being all red in the thermal images. However, when ∇*B_r_* is 0.35 mT mm^−1^, the magnetic field at the center of the working space is nearly zero and P_7_ is heated to a temperature of 45.6 °C (red color in the thermal image), while P_3_, P_6_, P_8_, and P_11_ show an orange color, with a mean temperature of 39 °C. Because the radial distance from P_7_ to P_2_, P_4_, P_10_, and P_12_ is larger, the magnetic field corresponding to them is higher and their color in the thermal image is slightly more yellow than orange, with an average temperature of 35 °C, excluding P_4_.

The remaining points show a yellow color in the thermal image and an average temperature of 32.3 °C. Finally, when ∇*B_r_* is increased to 0.65 mT mm^−1^, only P_7_ is heated to a temperature of 41.6 °C (red point in the thermal image), whereas the reaming points mostly remain in green color with an average temperature of 29.2 °C. In addition, for all the cases, the temperature at P_4_, shows a lower temperature when compared to the other points found at the same radial distance (P_2_, P_10_, and P_12_), indicating that the concentration of MNP was lower at that point. For the individual temperature at each point, refer to Section S6 in the Supporting Information.

From the previous experiments, we concluded that a gradient of 0.65 mT mm^−1^ can create an FFR that is sufficiently small to focus heating at any desired point containing MF2. As Figure 6D,F depicts, using the magnetic field produced by the Helmholtz coils Hx (*B_x_*) and Hy (*B_y_*), the position of the FFR and consequently the targeted point were changed. As the simulation and thermal images show, applying a magnetic field *B_x_* = −7.5 mT and *B_y_* = 7.5 mT, the FFR moves to P_2_, reaching a temperature of 44.7 °C, while the remaining points exhibited no significant temperature increase. Further, when *B_x_* = 15 mT, and *B_y_* = 0 mT, the FFR changes its position and focuses heating only to P_9_, reaching a temperature of 49.7 °C. Figure 6F shows the magnetic field distribution along the *X*‐axis for *B_x_* = 15 mT in a black line. Similarly, the graph shows the magnetic field when *B_x_* = −7.5 mT, and *B_y_* = 7.5 mT, for *y* = 11.5 mm in red, and for *y* = 0 in blue. As indicated by blue line, the magnetic field through the central line of points (P_5_ to P_9_) is above 8 kA m^−1^, which explains the reason no heating is observed in that line. However, for the second line of points (P_2_ to P_4_, at *y* = 11.5 mm) the magnetic field is zero at *x* = −11.5 mm, coinciding with the position of P_2_.

## 5 Actively Targeted Thermal Control of Magnetic Agents

**Figure 7:**
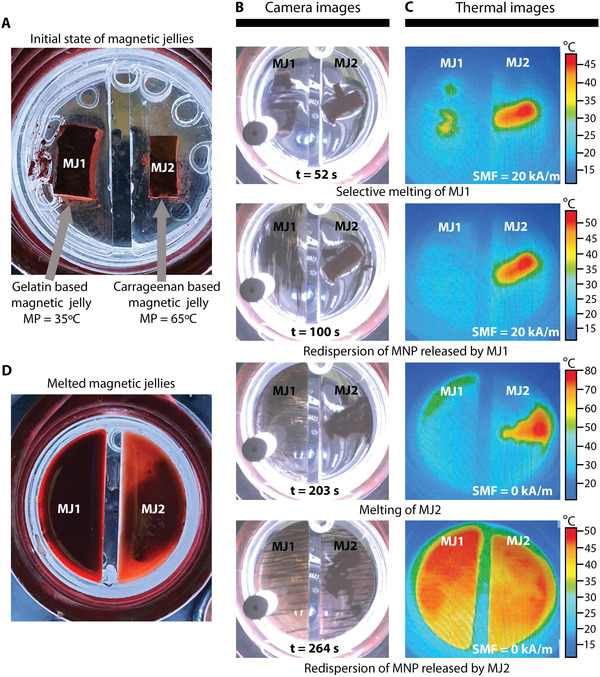
Selective melting of magnetic jellies. A) Gelatin‐based magnetic jelly (MJ1) and carrageenan‐based magnetic jelly (MJ2) inside the induction coil. Time‐lapse USB camera B) and thermal camera C) images of the selective melting of MJ1, the dispersion of the MNP released by MJ1, the melting of MJ2, and the dispersion of the MNP released by MJ2. D) Image showing the final state of MJ1 and MJ2 after the experiment.

To demonstrate certain potential applications of our system, we performed additional experiments combining the locomotion and heating control properties of magnetic millirobots and MNP. For the first experiment, two different magnetic jellies: a gelatin‐based jelly (MJ1) with a melting point (MP) of 35 °C, and a carrageenan‐based jelly (MJ2) with MP = 65 °C (see the Experimental Section), were used. We selectively melted first MJ1, while both jellies were exposed to the same AMF, by controlling the maximum temperature that could be attained via the application of an SMF, as shown in **Figure** **7**.

Figure 7A shows both magnetic jellies immersed in water within an acrylic container, inside the induction coil. Figure 7B,C shows the timelapse images captured using an USB camera and the timelapse thermal images of the jellies during the experimental procedure (see video S5 in the Supporting Information). When both jellies were exposed to an AMF of 16 kA m^−1^, without the influence of an SMF, MJ1 was torn apart and started to melt after 22 s, whereas MJ2 started to melt at ≈75 s as its melting temperature is higher.

To selectively melt MJ1, the maximum temperature that both jellies could reach needed to be controlled. For this, we applied an SMF of 20 kA m^−1^ while exposing MJ1 and MJ2 to an AMF. MJ1 melted 52 s after turning on the AMF, while the temperature of MJ2 increased to 45 °C. Subsequently, we dispersed the MNP previously released by MJ1 into the fluid by rotating the SMF, while MJ2 reached a maximum temperature of 54 °C, at *t* = 104 s. Further, on turning off the applied SMF, the temperature of MJ2 increased to ≈80 °C, completely melting and releasing the MNP by *t* = 203 s. Thereafter, we proceeded to disperse the MNP in the fluids containing the MNP released from MJ1 and MJ2, following which both dispersions reached similar temperatures (≈50°). This was expected because the MNP used in both jellies were the same.

**Figure 8:**
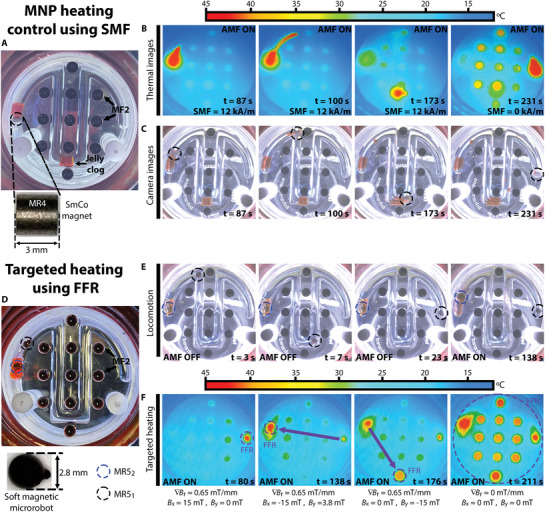
Locomotion and heating control. Experimental set A), timelapse USB camera B), and thermal camera C) image captures for the locomotion of a hard‐magnetic millirobot, the eddy current heating of electric conductive materials and suppression of the heating properties of soft‐magnetic materials using SMF. Experimental set D), timelapse USB camera E), and thermal camera F) images captures for the locomotion and targeted heat of soft‐magnetic millirobots using an FFR.

Then we tested the ability of MECS to simultaneously control the locomotion of magnetic millirobots and their heating properties through the application of an SMF and an FFR. First, we tested the locomotion of a hard‐magnetic millirobot (MR4) and its heating through eddy‐current losses, while suppressing the thermal properties of regions within the working space containing MF2 by the application of an SMF, as shown in **Figure** **8**A,C (see video S6 in the Supporting Information). Figure 8A shows the experimental set, which consists of an acrylic container with 11 holes containing MF2 and a circuit filled with silicone oil (*ν* = 2000 mm^2^ s^−1^). The circuit was blocked by two jelly clogs (gelatin jelly, MP = 35°), one placed right next to MR4 in its initial position, and one more in the middle of the circuit. As MR4 is a SmCo magnet, its electrical conductivity is sufficiently high to produce heat via eddy currents induced by the AMF

Figure 8B,C shows the timelapse thermal camera and USB camera images of the working space as the MR4 moved along the track to remove the clogs, while applying an SMF = 12 kA m^−1^ to suppress the heating properties of the regions containing MF2. The first capture in the time sequence shows the working space at *t* = 87 s, when MR4 successfully melts the first clog, while the thermal image shows that heating is focused only to the region surrounding MR4. Thereafter, MR4 begins to move toward the second clog (second frame, *t* = 100 s) while slightly heating the fluid that MR4 enters in contact with. At *t* = 173 s, MR4 removes the second clog, while the regions containing MF2 do not show any temperature increase. Finally, after the robot reaches its destination, we suppress the SMF and consequently, the temperature of the regions containing MF2 increases, showing a yellow color corresponding to an approximate temperature of 38 °C (*t* = 231 s).

Thereafter, we performed another experiment testing the locomotion and selective heating of two soft‐magnetic millirobots (MR5_1_ and MR5_2_) using an FFR, as shown in Figure 8D–F (see video S7 in the Supporting Information). Figure 8D shows the experimental setup, which comprises an acrylic container with 11 holes containing MF2, an unrestrained soft‐magnetic millirobot (MR5_1_), and a soft‐magnetic millirobot (MR5_2_) constrained within a magnetic jelly. The first three images of Figure 8E show the locomotion of MR5_1_ along the track till it reaches the targeted region at *t* = 23 s while the AMF is off, whereas the fourth image in the sequence shows MR5_2_ melting the jelly clog when an FFR (∇*B_r_* = 0.65 mT mm^−1^) is positioned at its location while the AMF is on. Further, Figure 8F shows time‐lapse thermal camera images at different points of the experimental procedure. The first thermal image shows the increase in heating of MR5_1_ after the AMF is turned on (at *t* = 60 s). Because the FFR is applied at the location of MR5_1_ (*B_x_* = 15 mT, *B_y_* = 0 mT), only the temperature of the millirobot MR5_1_ increases while the temperatures of the regions containing MF2 and the robot MR5_2_ do not increase. Subsequently, the FFR was shifted to the location of MR5_2_ by setting *B_x_* = −15 mT, and *B_y_* = 3.8 mT; hence, the temperature of MR5_1_ decreased while the temperature of MR5_2_ increased, as shown in the second thermal image, completely melting the jelly cloth by *t* = 138 s.

Thereafter, the FFR was shifted to a point containing MF2, located in the lowest region, by changing the magnetic field to *B_x_* = 0 mT, and *B_y_* = −15 mT, thereby reducing the temperature of MR5_2_ while increasing the temperature of the targeted MF2 (third thermal image, *t* = 176 s). Finally, when the FFR was turned off, the temperature of both the millirobots and all the points containing MF2 increased, as shown in the last frame of the thermal image sequence (*t* = 211 s).

## 6 Verification of MECS for Biomedical Applications

To exemplify the potential use of MECS for biomedical applications, two different experiments were performed: locomotion of biocompatible millirobot in an artificial body fluid (plasma fluid) and selective hyperthermia of NIH‐3T3 cells.

**Figure 9:**
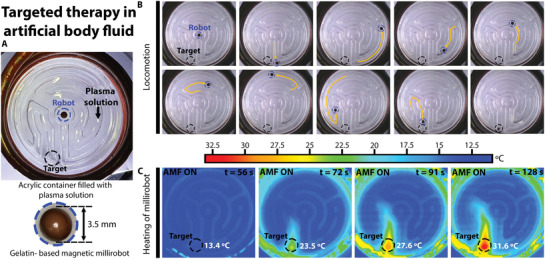
Targeted therapy in an artificial body fluid. A) Experimental set comprising an acrylic container with a labyrinth filled with plasma solution and a gelatin‐based magnetic millirobot. B) Captured images of the robot locomotion from the starting point to the targeted zone. C) Heating of millirobot at the targeted zone by application of an AMF.

**Figure** **9** shows the locomotion of a gelatin‐based magnetic millirobot (MR6) along a path of plasma solution (HK inno.N, Korea) toward a targeted zone, where the millirobot is heated by applying an AMF (see video S8 in the Supporting Information). Figure 9A shows the experimental setup comprising an acrylic container filled with the plasma solution, MR6, and the AMF coil. The trajectory of the robot toward the targeted point is shown through the captured images in Figure 9B. The current robot position at each photograph is marked with a blue circle; the translation done by the robot from the previous to the actual frame is shown using a yellow arrow, and the targeted point is denoted by a black circle. When the robot reached the targeted point, an AMF was applied to cause the temperature of the robot to rise from 13.4 °C (*t* = 56 s) to 31.6 °C (*t* = 128 s), as shown in Figure 9C. The temperature rise of MR6 caused the plasma surrounding it to increase its temperature as well.

**Figure 10:**
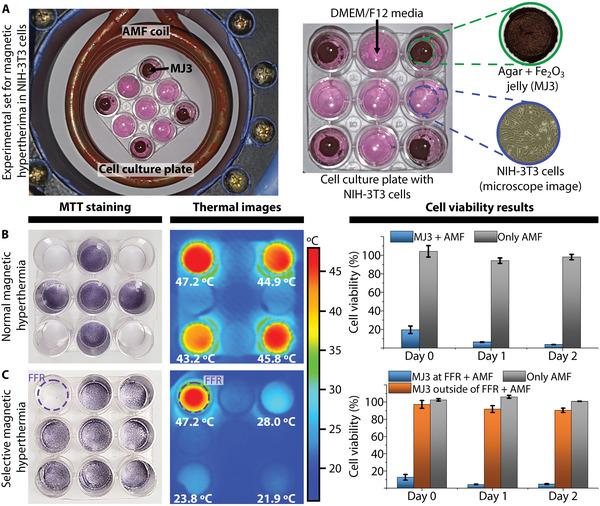
Selective magnetic hyperthermia. A) Experimental set comprising a 3 × 3 cell culture plate with NIH‐3T3 cells, DMEM/F12 media, agar‐based magnetic jelly, and coil system. MTT staining results, thermal images taken during the hyperthermia procedure, and cell viability results for normal magnetic hyperthermia B) and selective hyperthermia of NIH‐3T3 cells C).

To demonstrate the potential use of the system for improved magnetic hyperthermia, traditional and selective magnetic hyperthermia were performed in the NIH‐3T3 cells, as shown in **Figure** **10**. For this, the NIH‐3T3 cells were plated in 3 × 3 well plates with a density of 1 × 10^5^ cells per well and cultured overnight (see the Experimental Section). Out of the nine wells containing the NIH‐3T3 cells, agar‐based magnetic gelatin (MJ3) was placed at the four wells located at the vertex of the square‐shaped 3 × 3 well grid, as displayed in Figure 10A, for the application of magnetic hyperthermia. Two different hyperthermia conditions were applied during the experiments: normal hyperthermia, where just an AMF was applied to the culture plate, and selective magnetic hyperthermia, where a combination of SMF, FFR, and AMF was applied to selectively heat only one of the four wells containing MJ3.

The experiments were performed three times for each of the two experimental conditions for 10 min. Figure 10B,C shows MTT staining and cell viability results 2 days after hyperthermia, as well as thermal images captured while hyperthermia was being performed for normal hyperthermia (Figure 10B) and selective magnetic hyperthermia conditions (Figure 10C).

Thermal images show that, for normal hyperthermia, the temperature in all the wells containing MJ3 was between 43 and 47 °C, whereas that at the rest of the wells was below 22 °C. For selective hyperthermia, only the temperature at the targeted well increased significantly (47.2 °C), whereas that at the rest of the wells containing MJ3 was below 28 °C. Furthermore, the MTT staining results perfectly matched the thermal images for both hyperthermia conditions, showing no staining at the wells at which hyperthermia was applied.

The cell viability results for normal hyperthermia showed that the cell death rate of the wells containing MJ3 was ≈80. 5 ± 4.0% and 96.3 ± 0.08% at day 0 and day 2, respectively, while that of only the AMF stimulation (no MJ3) was less than 6%. In contrast, the cell viability results for selective hyperthermia showed that two days after hyperthermia, the cell death rate for the wells containing MJ3 located outside of the FFR 2 was equal to 9.5 ± 2.4%, whereas that of the well located at the FFR was about 95. 3 ± 3.3%. Notably, the cell death of the wells containing MJ3 and located outside of FFR was similar to that of the wells that had no MJ3, showing no significant toxicity of MJ3 or cell death due to the AMF. These results showed that, using MECS, the heating volume for magnetic hyperthermia applications can be controlled, leaving unharmed tissue outside the FFR, even when there is a significant amount of MNP located at the healthy tissue.

## 7 Discussion and Conclusion

In this study, we presented a novel MECS for the multimodal actuation of magnetic microrobots and control of the heating produced by soft‐magnetic millirobots and MNP. Most researchers use pure magnetic torque control, or dragging of magnetic microrobots using magnetic force; however, we demonstrated the automatic control of both types of locomotion in our system. The locomotion of magnetic swarms has garnered the attention of researchers of late. Thus, we implemented the 2D locomotion of a magnetic swarm in our system, by generating a magnetic TP, causing the millirobots composing the swarm to self‐assemble around the TP. Moreover, by varying the magnitude of the magnetic gradient distribution producing the TP, we could control the separation distance between the lead millirobot (located at the TP) and the orbiting millirobots. Further, by superpositioning a uniform magnetic field, we shifted the position of the TP, and as a result, achieved locomotion control of the magnetic swarm. This is the first report of a static coil system where TP locomotion has been observed. The locomotion experiments demonstrated that our system can create a wide combination of magnetic field distributions, which can control magnetic microrobots with different types of locomotion mechanisms or microrobots designed to operate by two or more locomotion mechanisms (multimodal locomotion). Furthermore, locomotion in our system is not limited to single millirobots, and locomotion of magnetic swarms was also demonstrated. In addition, the control of magnetic microrobots in our system is not limited only to the locomotion mechanisms presented in this paper; in fact, we believe that most of the existing mechanisms for the single and collective locomotion of magnetic microrobots can be implemented in our system. Further comparison with other electromagnetic systems is provided at the end of Section S2 in the Supporting Information.

The magnetic fields created by our system are not only useful for the actuation of magnetic microrobots but can also control the heating produced by soft‐magnetic microrobots and MNPs when exposed to high‐frequency AMF. We tested the influence of a time SMF in the heating of a noncoated iron oxide MNP ferrofluid (MF1) and an oleic acid‐coated iron oxide MNP fluid (MF2). The application of the SMF decreased the maximum temperature that both fluids could attain, requiring smaller values of SMF for MF2 due to its higher diameter and individual coating, which allowed the fluid to follow a superparamagnetic behavior close to that described via Langevin equations for a MNP with a core size of 25 nm. In addition, through our experiments, we also proved that our system can control the maximum temperature that any particular soft‐magnetic material can achieve, which is useful for magnetic hyperthermia and drug release applications. This was demonstrated by the selective melting of MJ1, while controlling the maximum temperature of MJ1 and MJ2. Although not demonstrated in this study, the implementation of an SMF can also enhance the thermal properties of the MNPs.

Using a pair of air‐core Maxwell coils we created a region with zero magnetic field at the center of the coils, and a linearly increasing magnetic field in all directions, which increasingly magnetized the MNP as they moved away from the center, till they reached their magnetic saturation. The ability to release heat of MNP is suppressed when MNPs are saturated; thus, only unsaturated MNPs close to the center of the maxwell coils (i.e., those within the FFR) can release heat. Consequently, using the FFR in superposition with an SMF produced by the pairs of Helmholtz coils, we controlled the diameter of the targeted area and its position, being able to focus heat selectively within a small area with a diameter of 5 mm. In contrast to the few previous systems that have achieved focused heating, this is the first system to achieve dynamic control of the heating volume, and the first to achieve control of the FFR position without mechanical mechanisms as well. This was possible because the implementation of the pair of coreless maxwell coils, which, although require more electric power, provided a bigger working space while the coils themselves occupied a smaller volume compared to magnets or iron‐core electromagnets.

We also demonstrated the ability of our system to simultaneously implement locomotion and heating applications. First, we used a SmCo magnet millirobot (MR4) for locomotion and thermal ablation of jelly clogs, using eddy‐current‐induced heating, while suppressing the heating properties of all regions filled with MF2 by using an SMF. This experiment demonstrated that our developed system can control the locomotion and heating of devices designed for eddy‐current thermal ablation applications,^[^ ^49^, ^50^ ^]^ while combining it with the use of functionalized MNPs or fluids for the tracking of the instruments through magnetic particle imaging, or other applications requiring the use of MNPs and preventing the MNPs from heating unwanted regions. Finally, in the last experiment, we demonstrated the locomotion and selective heating of two soft‐magnetic millirobots (MR5_1_ and MR5_2_). First, we controlled the locomotion of MR5_1_ through a designed path and thereafter, using an FFR, the heating properties of the regions containing MF2 and MR5_2_ were suppressed, heating only the millirobot MR5_1_. Subsequently, the position of the FFR was changed to MR5_2_ and the gelatin clog melted, wherein MR5_2_ was constrained. This demonstrated that our proposed system can simultaneously control the locomotion and heating properties of magnetic agents.

The potential use of MECS for biomedical applications was verified by the locomotion of a gelatin‐based magnetic millirobot, which was driven through a labyrinth filled with plasma fluid. After reaching the targeted zone, the temperature of the millirobot was increased by the application of an AMF. Furthermore, selective magnetic hyperthermia therapy using MECS was demonstrated by selectively heating one of the four wells in the plate containing NIT‐3T3 and an agar‐based magnetic jelly (MJ3). When normal hyperthermia was applied (no application of an FFR), the survival rate of the cells in the wells containing MJ3 was below 3.7%; in contrast, when selective hyperthermia was applied (application of an FFR), the death rate of such well was ≈95.3%. Through these experiments, heating was successfully focused in a circular area with a diameter of 1 cm containing the NIT‐3T3 cells. However, the use of better MNP or MNP should improve the resolution. For the successful application of selective magnetic hyperthermia, more detailed studies are required to quantify the heat produced by the MNP under different conditions (dispersed in fluid, fixed in jelly, and forming magnetic assembles) in accordance with their magnetization values and the heat propagation from the targeted tissue toward the surrounding tissue to establish a reliable protocol and control algorithm for selective magnetic hyperthermia.

In its current condition, the developed system can be used for in vitro applications. However, for in vivo scenarios, the system still requires an additional imaging system (e.g., MRI, ultrasound) to track the magnetic agents. Therefore, it would be difficult to adapt to the existing medical imaging systems for such purposes without a significant modification to such systems or our developed electromagnetic system. However, the experimental magnetic particle imaging technology uses the same physical principles as the ones used in this article for selective magnetic hyperthermia, as well as the same FFR, SMF, and AMF magnetic fields. Therefore, just by adding a pick‐up coil in our system, the magnetic imaging particles could be implemented and used to track magnetic robots and monitor their temperatures to implement closed‐loop control of theranostic agents. In our future work, we will work toward the development of a theranostic system by implementing magnetic particle imaging in our MECS, as well as exploring in detail the spatial and temporal resolution for selective magnetic hyperthermia based on different properties of MNP.

## 8 Experimental Section

### Experimental Set

Images of MECS, power supplies and the full specifications of the coils have been presented in section S2. For all the experiments, the thermal pictures were acquired using a thermal camera Testo 875 (Testo, UK), and the thermal videos were obtained using a USB webcam (QC4K, QSENN) to visualize the screen of the thermal camera in the user interface and record video. Further, to record the locomotion of magnetic millirobots, a Dino‐Lite AM4113FVT USB microscope camera (Dino‐Lite, Taiwan) and a Dino‐Lite AM73915MZTL USB microscope camera (Dino‐Lite, Taiwan) were used. For experiments on MR2, MR3, and MS4, the camera AM4113FVT was used to record the *XZ* plane, while the *XY* plane was recorded using the camera AM73915MZTL. For the rest of the experiments, AM73915MZTL was placed on the *ZX* plane and AM4113FVT on the *XY* plane.

### Magnetic Millirobots Fabrication

For the experiments, six different magnetic millirobots (MR1 to MR6) and a magnetic swarm (MS1) were designed. MR1 is a neodymium N35 ball magnet, with a diameter of 5 mm, painted in red to facilitate image processing. MR2 was manufactured using a DLP 3D printer (Micro Plus Advantage, Envisiontec, USA), having of a spherical body with a diameter of 3 mm and containing a Neodymium N35 magnet with diameter and length of 0.5 and 2 mm, respectively. The spherical body comprised two helicoidal tails of radius 1 mm each, resulting in a total body length of 10 mm. The robot was painted in red to facilitate image processing.

MR3 was fabricated by cutting a circle of 1 mm diameter of black acrylic having a thickness of 2 mm and a hole at its center of radius 0.5 mm and depth of 1 mm to place a disk neodymium magnet with the same characteristics. Further, MS1 was made by using several MR3 robots, using black acrylic only for the lead magnet, and blue acrylic for the remaining millirobots comprising the magnetic swarm. MR4 is a cylindrical SmCo magnet, with a diameter and height of 3 mm each.

To make MR5, a photocurable resin (Form 1+ clear V4, Formlabs, USA) was mixed at 70 wt% with Fe_2_O_3_ MNP (20 nm, US Research Nanomaterials, Inc., USA) at 30 wt%, and stirred by hand for 15 min. Thereafter, the solution was placed inside a syringe and injected into a container with silicon oil (*ν* = 100 000 mm^2^ s^−1^) while exposed to ultraviolet light. For a detailed image of each millirobot, refer to Section S3 in the Supporting Information. A similar procedure was used for the fabrication of MR6, but the resin was substituted by gelatin at 10 wt%, which was injected into the container with silicon oil and subsequently placed inside a fridge to complete the gelation. Thereafter, MR6 was washed with distilled water to remove any oil remaining at the surface of the robot.

### Preparation of MF2

We bought oleic acid coated MNP dispersed in chloroform with an iron concentration of 25 mg mL^−1^ (Ocean Nano Tech, USA). However, the boiling point of chloroform is 61.2 °C, making it unsuitable for heating tests because it could easily evaporate during the experiments; thus, the solvent to decane (boiling point 174.1 °C) was changed. To change the solvent to decane, first, 4 mL of the original fluid was taken and it was placed in a vacuum oven at an absolute pressure of 30 kPa and a temperature of 60 °C to dry it slowly overnight. Thereafter, 2 mL of decane (Decane, Anhydrous, ≥99%, Sigma‐Aldrich) was added and the MNP was dispersed by 5 min sonication bath. Although the iron concentration was intended to be of 50 mg mL^−1^, certain amount of the MNP precipitated to the bottom of the container; thus, the real concentration was slightly lower.

### Preparation of Magnetic Jellies

Magnetic jelly 1 (MJ1) was prepared by mixing gelatin (Duksan, Korea) at 10 wt%, Fe_2_O_3_ MNP (20 nm, US Research Nanomaterials, Inc., USA) at 30 wt% with distilled water inside a 20 mL vial. The solution was stirred using a magnetic stirrer at 60 °C for 2 h. Then 6 mL of the solution was poured into a 16 mm × 26 mm rectangular plastic mold, and stored in a refrigerator. Similarly, Magnetic jelly 2 (MJ2) was prepared by mixing carrageenan (*ι*‐carrageenan, Sigma‐Aldrich) at 4 wt%, Fe_2_O_3_ MNP at 30 wt% with distilled water inside a 20 mL vial. The solution was stirred using a magnetic stirrer at 85 °C for 8 h. Then 6 mL of the solution was poured into a 16 mm × 26 mm rectangular plastic mold, and stored in a refrigerator. Magnetic jelly 3 (MJ3) was prepared by mixing Fe_2_O_3_ MNP at 30 wt% and sterilized agar at 2 wt% in distilled water inside a 40 mL vial. The solution was stirred using a magnetic stirrer at 85 °C for 2 h. Subsequently, 6 mL of the solution was poured into a 60 mm petri dish (SPL Life Science. Korea), and stored in a refrigerator.

### Cell Cultivation and Survival Analysis

NIH‐3T3 cells (Korea Cell Line Bank, Seoul, Republic of Korea) were cultured in Dulbecco's modified Eagle's medium/Ham's F‐12 50/50 (DMEM/F12; Gibco, New York, NY, USA), and supplemented with 10% fetal bovine serum (FBS; Gibco, Grand Island, NY, USA), 2 × 10^−3^ m l‐glutamine, 100 U mL^−1^ penicillin, and 100 µg mL^−1^ streptomycin (Gibco, Carlsbad, CA, USA) in a humidified incubator at 37 °C with 5% CO_2_. The medium was changed every other day.

The cell viability was measured by CCK‐8 (CCK‐8; Dojindo Molecular Technologies, INC. Korea) assay kit using a microplate reader with absorbance at 450 mm at 0, 1, and 2 days after hyperthermia. The metabolically active cells in 48‐well after stimulation were determined by MTT staining. At 2 days of hyperthermia, media were replaced with fresh DMEM/F12 containing MTT (Thiazolyl Blue Tetrazolium Bromide, Sigma‐Aldrich) solution in PBS (2 mg mL^−1^) for 4 h.

### Statistical Analysis

All experiments were performed at least three times with similar results. Results in all graphs are presented as mean values ± standard error of the mean (SEM).

## Conflict of Interest

The authors declare no conflict of interest.

## Supporting information

Supporting Information

Supplemental Video 1

Supplemental Video 2

Supplemental Video 3

Supplemental Video 4

Supplemental Video 5

Supplemental Video 6

Supplemental Video 7

Supplemental Video 8

## Data Availability

The data that supports the findings of this study are available in the supplementary material of this article.
