# Supplementary material for: Multimodal Locomotion and Active Targeted Thermal Control of Magnetic Agents for Biomedical Applications
Source: Adv Sci (Weinh). 2022 Jan 20;9(7):2103863. doi: 10.1002/advs.202103863 (PMC8895130; doi:10.1002/advs.202103863)
Supplement: Supplementary file 1 — Supporting Information [file ADVS-9-2103863-s007.pdf]

ADVANCED SCIENCE
Open Access

Supporting Information

for *Adv. Sci.*, DOI: 10.1002/advs.202103863

Multimodal locomotion and active targeted thermal control of magnetic agents for biomedical applications

*Armando Ramos-Sebastian, So-Jung Gwak, and Sung Hoon Kim**

Supporting Information

Multimodal locomotion and active targeted thermal control of magnetic agents for biomedical applications

*Armando Ramos-Sebastian, So-Jung Gwak, and Sung Hoon Kim**

Section 1. Selective magnetic hyperthermia

As described in **Figure S1**, under a high-frequency alternating magnetic field (AMF) all the regions with MNP experience an increase in temperature. However, when applying the AMF in combination with an FFR, only the temperature of the MNP found at the center of the FFR increases.

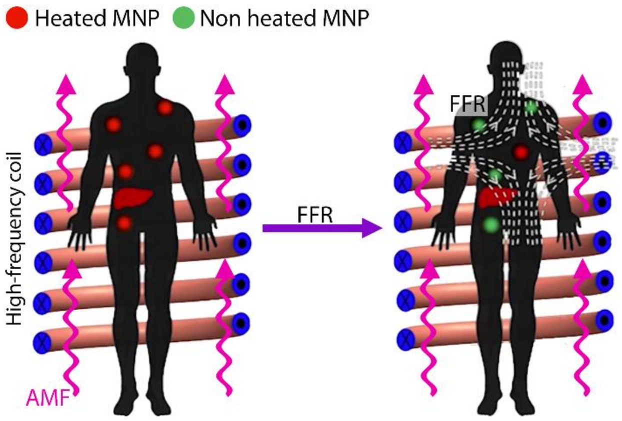

Figure S1. Selective magnetic hyperthermia.

Section 2. Detailed configuration of MECS

For the design of our system, we used a commercial induction heating coil (Hc) (Osung Hitech, OSH-R5), comprising a 5 kW power supply and a solenoid coil with the number of turns N = 4 and a diameter D = 6.5 cm. The Hc coil control system had a fixed frequency of 200 kHz, and only the ON/OFF and power (in percentage) could be manually controlled. To obtain the amplitude of the magnetic field, we used a pick-up coil with N = 7 and D = 2 cm to measure it indirectly using Faraday's law of induction, as shown in **Figure S2**.

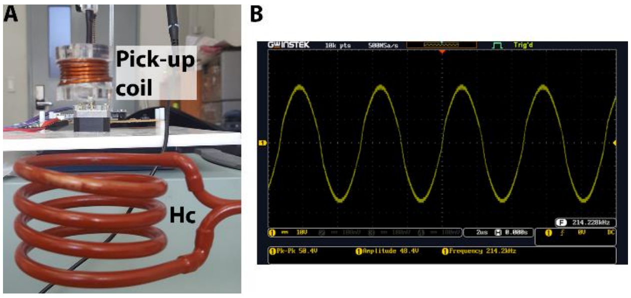

Figure S2. Measuring the magnetic field of Hc. (A) Experimental set. (B) Induced voltage on pick-up coil.

Through the measurements, we obtained the following equation to control the amplitude H_{AMF} of the alternating magnetic field (AMF):

$$H_{AMF} = 0.0018P^2 + 0.1672P + 2.5275 \quad \text{(S1)}$$

where P is the power supplied from the power supply in percentage. Considering the volume that can fit within the coil Hc, we designed the remaining coils for a working space of 60 mm x 60 mm x 60 mm. First, the pair of Maxwell coils are designed because they are in charge of creating the field-free region (FFR). They require a high current for generating a gradient above 2 mT mm^{-1}, which is the minimum gradient typically reported for magnetic particle imaging applications and available in commercial magnetic particle imaging systems (24). We designed our pair of Maxwell coils for a magnetic gradient field of **G** = [-1.5 -1.5 3] mT mm^{-1}.

Table S1. Coil's specifications

Coil	N	D [cm]	R [Ω]	L [mH]	B_{AC} [mT]	I_{AC} [kW]	P_{AC} [kW]	B_{DC} [mT]	G [mT mm^{-1}]	I_{DC} [A]	P_{DC} [kW]
Hc	4	6.5	/	/	27.6	445	5	/	/	/	/
Mc	200	14	0.47	5.3	/	/	/	/	3	100	10
Hz	583	28	4.47	139.5	37.4	10	1	93.5	0.32	25	3
Hx	430	44	3.78	130	52.8	30	3	52.8	0.24	30	3
Hy	602	60	7.51	361	27	15	3	54	0.18	30	10

Thereafter, we designed the pairs of Helmholtz coils in the following order: Helmholtz coils for the Z-axis (Hz), Helmholtz coils for the X-axis (Hx), and Helmholtz coils for the Y-axis (Hy). The pairs of Helmholtz coils were designed to produce a magnetic field **B** = [45 45 90] mT, which is sufficiently high for moving the FFR along the working space. Because all of our coils were designed with an air core, to obtain the designed magnetic fields high values of electric current were required. Further, to keep the coils at a temperature below 20 ºC, we designed all the coils (except for Mc) to fit within non-magnetic closed steel containers such that they could be cooled using a chiller (SJ-20AS, Samjung, Korea). The case for Mc was made of brass, because it had a lower electrical conductivity than steel, and thus, it could be placed close to the induction coil.

Table S1 shows the number of turns (N), diameter (D), electric resistance (R), inductance (L), maximum AC magnetic field (B_{AC}), maximum AC electric current (I_{AC}), maximum AC electric power (P_{AC}), maximum DC magnetic field (B_{DC}), maximum DC electric current (I_{DC}), maximum gradient magnetic field (G), and the maximum DC electric power (P_{DC}) of each individual coil of the system.

To move the FFR throughout the working space, we required approximately 10 kW to power each of the Hy coils. However, there was no AC/DC power supply of 10 kW available in our laboratory; therefore, we used a combination of AD/DC power supplies and DC power supplies to power our system. Magnetic contactors (LC1DT40BL) were used to interconnect the coils and the power supplies based on the required function for the system: locomotion and heat control through SMF, or heat control using SMF and FFR. For locomotion and heat control, the Hz coils were each connected to an APS-1102A AC/DC power supply (10 A, 1 kW, GW Instek, Taiwan), whereas all the coils of Hy and Hz were each connected to an APS-2302 AC/DC power supply (30 A, 3 kW, GW Instek, Taiwan). Further, for heat control using SMF and FFR, all the coils of Hz and Hx were each connected to an APS-2302 AC/DC power supply, while

the coils of Hy were connected to an EX300-32 DC power supply (10 kW, ODA Technologies, Korea). Furthermore, the coils in Mc were controlled by an N8926A DC power supply (10 kW, Keysight, USA) each. The complete system is shown in **Figure S3** and **Figure S4**.

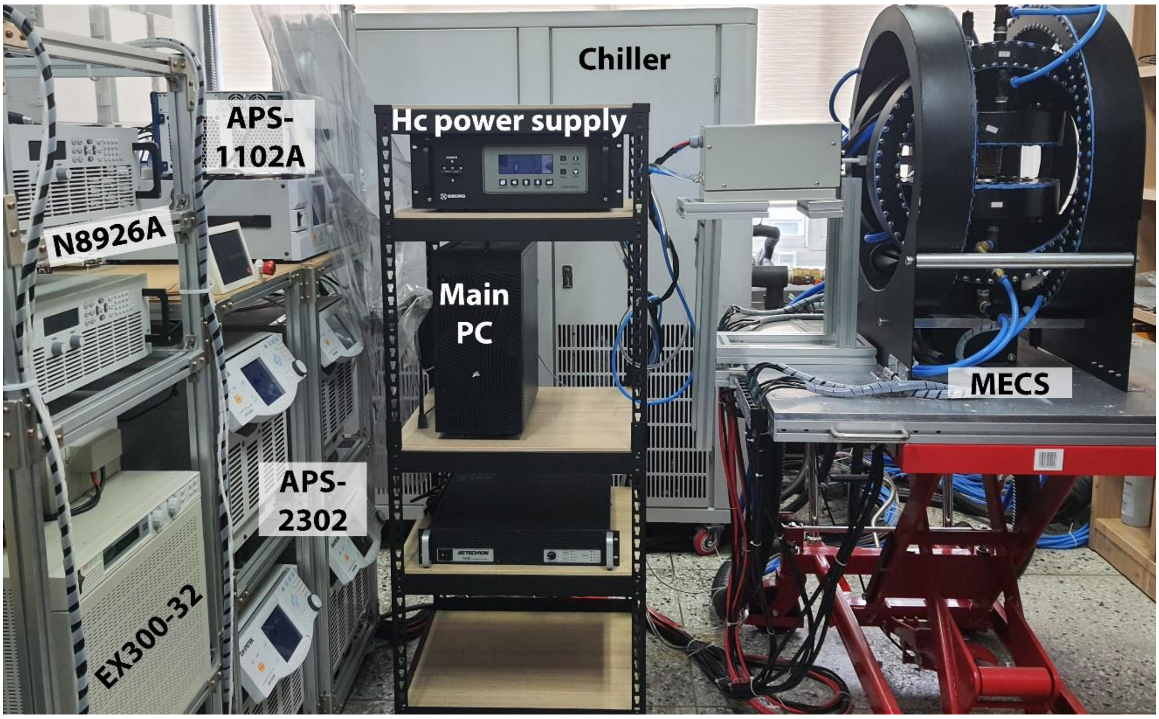

Figure S3. MECS.

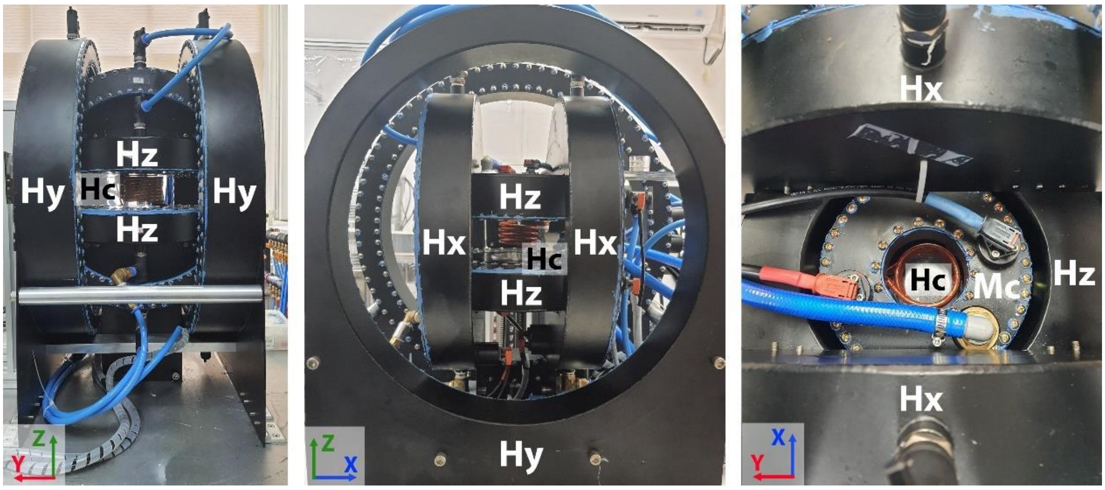

Figure S4. Different planes of MECS.

The magnetic field produced by the three pairs of Helmholtz coils is:

$$\mathbf{B} = \begin{bmatrix} B_x \\ B_y \\ B_z \end{bmatrix} = \left(\frac{4}{5}\right)^{3/2} \mu_0 \begin{bmatrix} n_x I_x / R_x \\ n_y I_y / R_y \\ n_z I_z / R_z \end{bmatrix} \quad \text{(S2)}$$

where n, I, and R are the number of turns, electric current, and radius of each coil, respectively. Because n and I are constants, we can simplify the expression as:

$$\mathbf{B} = \begin{bmatrix} k_x\, I_x & k_y\, I_y & k_z\, I_z \end{bmatrix}^{\mathrm{T}} \tag{S3}$$

where k_x, k_y, and k_z are constants that depend on the properties of the coils. The magnetic field created by the pair of Maxwell coils is:

$$\mathbf{B} = I_m \mathbf{G}\, \boldsymbol{p} \tag{S4}$$

where **G** is the 3 x 3 diagonal gradient matrix:

$$\mathbf{G} = k_m\ diag[\ 1/2,\ 1/2,\ -1] \tag{S5}$$

where k_m is a constant that is dependent on the coil geometrical properties. All the constants k_x, k_y, k_z, and k_m can be calculated; however, for consistency between our simulations and the real magnetic field distribution, we obtained them experimentally.

Using a gaussmeter (5180, F.W. Bell, USA), we measured the magnetic field produced by the Helmholtz coils at its center for different values of electric current. Similarly, we measured the magnetic field produced by the pair of Maxwell coils along the Z and X axis and calculated its magnetic gradient, as shown in **Figure S5**. The gradient produced in the X axis is the same as that in the Y axis and the gradient produced along any radial axis perpendicular to the Z axis will be same. Thus, we can describe the gradient distribution only through two terms $\mathbf{G}_z$ and $\mathbf{G}_r$ ($\mathbf{G} = diag[\mathbf{G}r,\ \mathbf{G}r,\ -\mathbf{G}z]$). We compared the measured data with the simulated values and slightly modified the simulation parameters such that the simulated values matched the measured ones, to enable higher precision when calculating the FFR. Through these measurements and simulations, we determined the following values: $k_x = 1.76\ x\ 10^{-3}$, $k_y = 1.8\ x\ 10^{-3}$, $k_z = 3.74\ x\ 10^{-3}$, and $k_m = 0.035$.

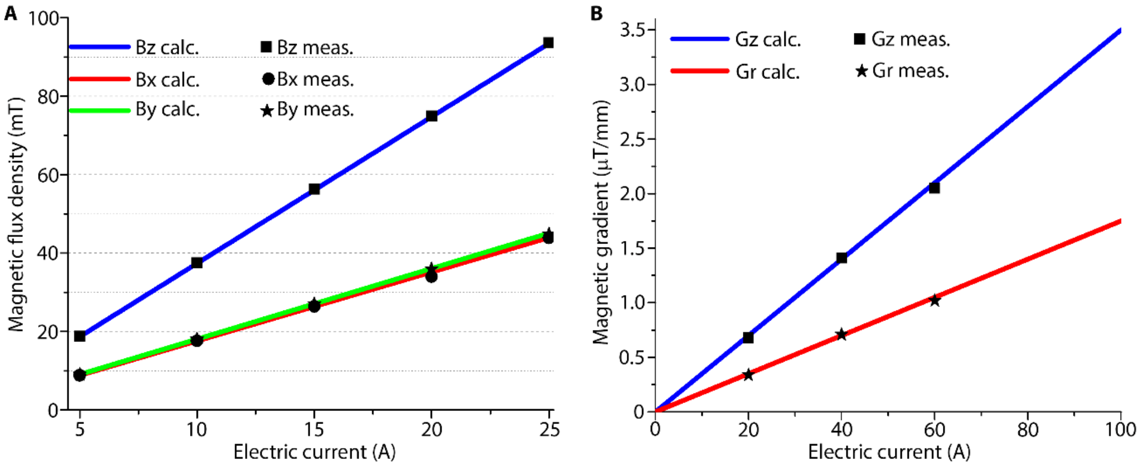

Figure S5. Magnetic field characterization. A) Simulated and measured magnetic fields produced by the pairs of Helmholtz coils. B) Simulated and measured magnetic fields produced by the pairs of Maxwell coils.

To better illustrate the improvements in MECS over existing electromagnetic coils systems, **Table S2** summarizes the differences of other systems comprising pairs of Helmholtz and Maxwell coils for the control of magnetic microrobots. When comparing MECS even with the recently reported systems (2020-2021), it can be observed that MECS has the biggest working space, produces the highest uniform magnetic fields and magnetic field gradients, and implements close-loop multimodal locomotion of single magnetic microrobots; it is the only system that can produce magnetic traps for the self-assembly and control of magnetic swarms. Moreover, it performs temperature control of MNP and selective heating of MNP.

Table S2. Comparison of MECS with other electromagnetic systems

Reference	Type of Coils	Type of fields	Working space	Type of locomotion	Heating control
[1] 2012	2 pairs of Helmholtz coils, each pair with one power supply.<br>2 pairs of Maxwell coils, each pair with one power supply.	2D uniform magnetic fields up to 3.5 mT<br>Gradients up to 0.12 mT/mm	Square<br>30 mm × 30 mm	No locomotion was demonstrated	Not possible.
[2] 2013	3 pairs of Helmholtz coils, each pair with one power supply.<br>1 pairs of Maxwell coils, with one power supply.	3D uniform magnetic fields, max. value not specified<br>1 DOF Gradients, max. value not specified	Prismatic<br>20mm × 10mm × 20mm	3D Open-loop gradient force demonstrated in a simple trajectory.	Not possible.
[3] 2020	3 pairs of Helmholtz coils, each pair with one power supply.<br>3 pairs of Maxwell coils, each pair with one power supply.	3D uniform magnetic fields, max. value not specified(based on controller below 34 mT)<br>3D Gradients, max. value not specified(based on controller below 0.77 mT/mm)	Cubic<br>25mm × 25mm × 25mm	3D Closed-loop gradient force demonstrated in a simple trajectory.	Not possible.
[4] 2020	3 pairs of Helmholtz coils, each pair with one power supply.	3D uniform magnetic fields, reported 15 mT.<br>3D Gradients, reported 0.2 mT/mm.	Cubic<br>50mm × 50mm × 50mm.	3D Closed-loop gradient force demonstrated.	Not possible
[5] 2021	3 pairs of Helmholtz coils, each pair with one power supply.	3D uniform magnetic fields, reported 45 mT.<br>1 DOF Gradients, reported 0.32 mT/mm.	Cubic<br>50mm × 50mm x 50mm.	3D Closed-loop gradient force demonstrated.	Not possible
MECS	3 pairs of Helmholtz coils, with each coil independently controlled with one power supply.<br>1 pair of Maxwell coils, each coil independently controlled with one power supply.	3D uniform magnetic fields up to 93.5 mT.<br>3D Gradient magnetic fields up to 3 mT/mm.<br>High-frequency alternating magnetic field up to 27.6 mT.	Cylindrical, radius of 60 mm and height of 50 mm.	3D Close-loop Multimodal locomotion of single microrobots: torque-based locomotion, force-based locomotion.<br>2D locomotion using trapping points.<br>2D self-assembly and control of magnetic swarms	Temperature control of MNP temperature.<br><br>Selective heating of MNP, controlling the volume size and position.

Section 3. Magnetic microrobots

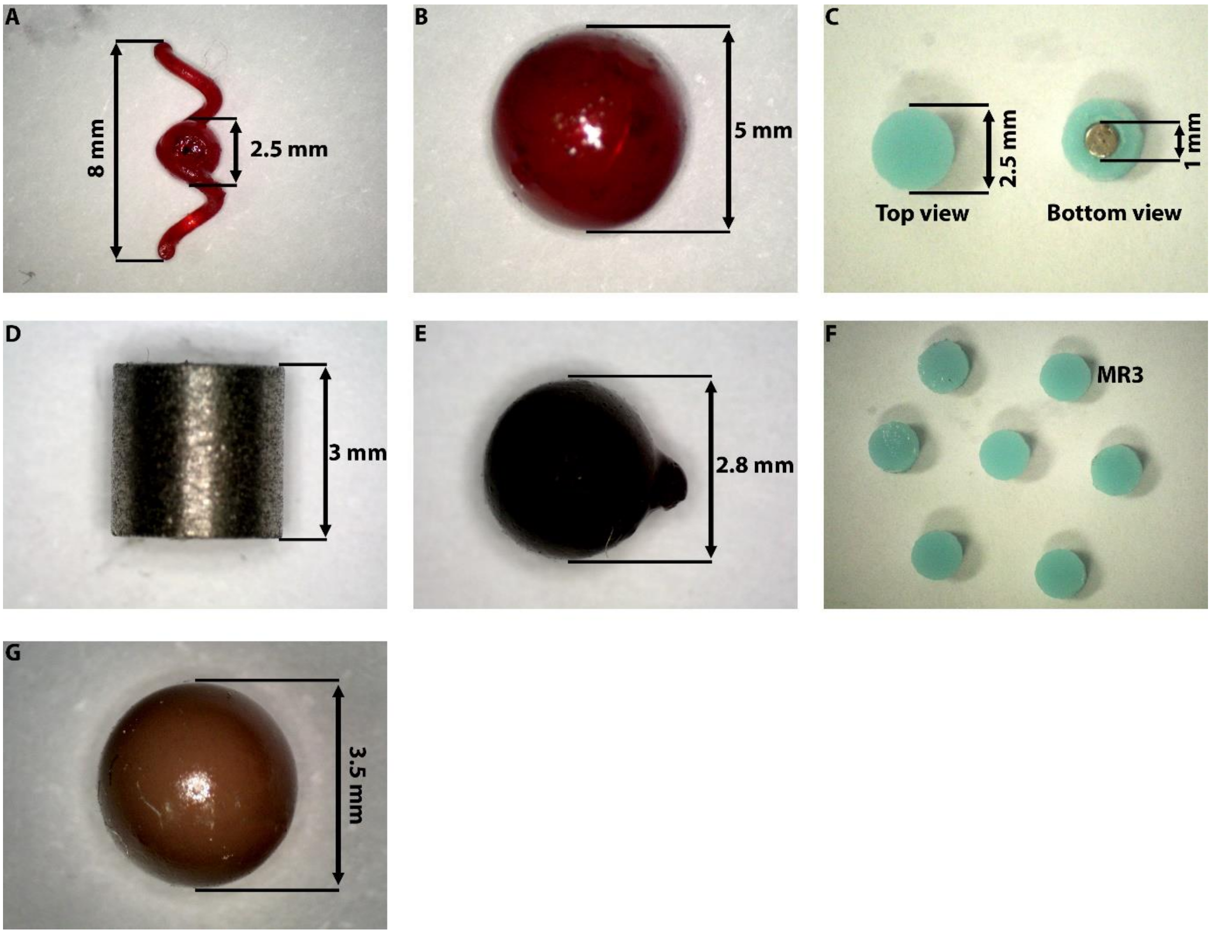

Figure S6. Designed magnetic microrobots. A) MR1. B) MR2. C) MR3. D) MR4. E) MR5. F) MS1. G) MR6

Figure S6 shows all the magnetic microrobots designed for the experiments. MR1 (Figure S6A) was used to test the locomotion of helicoidal microrobots through the application of uniform rotating magnetic fields. For magnetic force locomotion, we used robot MR2 (Figure S6B). As shown in Figure S6C, for M3 we placed a neodymium magnet inside an acrylic disk and used it for locomotion based on the trapping point mechanism. Further, for the locomotion and eddy-current heating experiments, we used MR4 (Figure S6D). MR5 was used for the locomotion and heating control by FFR experiments. In addition, the magnetic swarm MS1 (Figure S6F) was composed of seven identical MR3 robots. MR6 (Figure S6G) was used for the exemplification of targeted therapy and locomotion in an artificial body fluid (plasma fluid).

Section 4. Modeling for magnetic locomotion

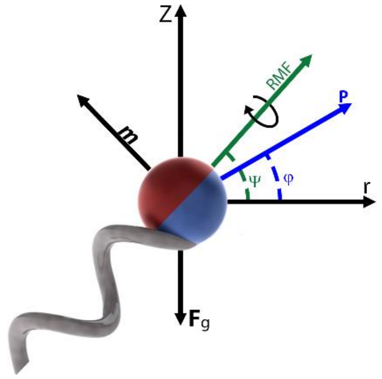

Figure S7. Forces acting on a helicoidal magnetic microrobot.

The magnetic torque exerted in a magnetic microrobot is:

$$\tau = \mathbf{m} \times \mathbf{B} \tag{S6}$$

where, $\mathbf{m} = \begin{bmatrix} \mathbf{m}_x & \mathbf{m}_y & \mathbf{m}_z \end{bmatrix}$ is the magnetic moment of the microrobot. To control the locomotion of a helicoidal magnetic microrobot, a rotation perpendicular to the helix axis has to be exerted in the microrobot through the implementation of a rotating magnetic field (RMF). The RMF is controlled using the following equation:

$$\begin{bmatrix} \mathbf{B}(t)_x \\ \mathbf{B}(t)_y \\ \mathbf{B}(t)_z \end{bmatrix} = B \begin{bmatrix} \cos\theta \sin\psi \sin(\omega t) - \sin\theta \cos(\omega t) \\ \sin\theta \sin\psi \sin(\omega t) + \cos\theta \cos(\omega t) \\ \cos\psi \sin(\omega t) \end{bmatrix} \tag{S7}$$

where θ is the azimuthal angle, and Ψ is the polar angle of the axis of the RMF. Assuming that the RMF has a sufficiently large magnitude such that the step-out frequency of the microrobot for a particular fluid is beyond the working range, the thrust force $\mathbf{F}_T$ in a helicoidal magnetic microrobot is proportional to the angular frequency ω; thus, we obtain

$$\mathbf{F}_T = \lambda \omega \tag{S8}$$

where λ is a constant that depends on the geometric properties of the robot and the fluid. Considering that the robot experiences a gravity force, $\mathbf{F}_g$, that causes it to move to the bottom of the container, to make the robot maintain its position, we must insert a compensation thrust force $\mathbf{F}_{Tg}$. Consequently, for the robot to swim in any desired direction φ (of the zr plane) an additional locomotion thrusting force $\mathbf{F}_L$ must be produced. Hence, the propulsion **P** is expressed as

$$\mathbf{P} = \begin{bmatrix} \mathbf{F}_{Tg} + \mathbf{F}_{Lz} \\ \mathbf{F}_{Lr} \end{bmatrix} = \begin{bmatrix} \lambda(\omega_0 + \omega_l)\sin(\psi) \\ \lambda(\omega_0 + \omega_l)\cos(\psi) \end{bmatrix} \tag{S9}$$

where ω_0 is the angular frequency at which the robot levitates in the fluid, ω_L is the angular frequency that produces the locomotion thrust, and Ψ is the orientation of the RMF. Solving for Ψ, we have

$$\psi = \arctan\left(\frac{\mathbf{F}_{Tg} + \mathbf{F}_{Lz}}{\mathbf{F}_{Lr}}\right) = \arctan\left(\frac{\mathbf{F}_{Tg} + \mathbf{F}_L \sin\varphi}{\mathbf{F}_L \cos\varphi}\right) \tag{S10}$$

then knowing Ψ, we can calculate ω as

$$\omega = \sqrt{\left(\left(\omega_0 + \omega_l\right)\sin\left(\psi\right)\right)^2 + \left(\left(\omega_0 + \omega_l\right)\cos\left(\psi\right)\right)^2} \tag{S11}$$

λ and ω_0 can be calculated using a set of complex equations, but the results may vary significantly from the real results. Hence, it is better to determine λ and ω_0 experimentally, which can be easily done using Equation S1. Thereafter, the user can control the locomotion of the microrobot by simply indicating the desired locomotion direction (φ, θ), force, and speed or locomotion frequency (f). Subsequently, the control algorithm calculates the required total angular frequency of the magnetic field as well as its rotation axis.

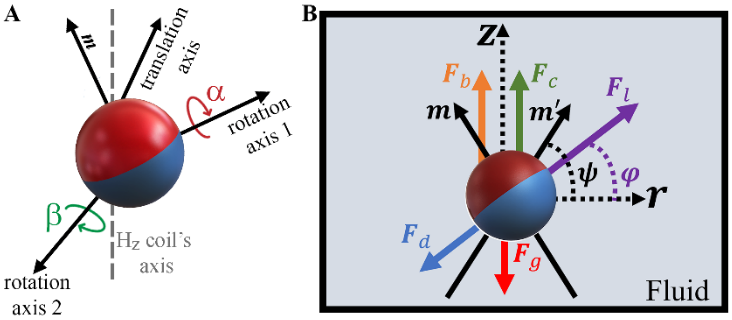

Figure S8. Magnetic force locomotion. A) Locomotion mechanism using magnetic force. B) Forces acting on the magnetic microrobot.

For the magnetic force locomotion, we used the pair of coils Hz to create a three-dimensional 1 DOF magnetic gradient field, as shown in **Figure S8**A, which drags the microrobot along the mirrored axis of the magnetic moment of the robot with respect to the axis of the coils Hz. Figure S8B shows the forces that act on the microrobot as it moves through a fluid. The microrobot experiences a force $\mathbf{F}_g$ due to gravity, a buoyancy force $\mathbf{F}_b$ that causes the magnet to move to the surface of the liquid, and a drag force $\mathbf{F}_d$ that opposes the movement of the microrobot in the fluid. We define $\mathbf{F}_c$ and $\mathbf{F}_l$ as the gravity compensation and magnetic locomotive forces, respectively, produced by the coil system. The magnetic force exerted on a microrobot by the pair of gradient coils is

$$\mathbf{F}_m = (\mathbf{m} \cdot \nabla)\mathbf{B} \tag{S12}$$

Because of the geometry of the gradient produced by the Hz coils, we analyze the magnetic

force in the ZR plane, where z is the vertical axis and r is any axis perpendicular to z. Redefining **m** as $\mathbf{m} = [\mathbf{m}_r \quad \mathbf{m}_z]$ we obtain

$$\mathbf{F}_m = \begin{bmatrix} \mathbf{m}_r \dfrac{\delta \mathbf{B}_r}{\delta r} + \mathbf{m}_z \dfrac{\delta \mathbf{B}_r}{\delta z} \\ \mathbf{m}_r \dfrac{\delta \mathbf{B}_z}{\delta r} + \mathbf{m}_z \dfrac{\delta \mathbf{B}_z}{\delta z} \end{bmatrix} \tag{S13}$$

As there are no electric currents in the working space, $\nabla \times \mathbf{B} = 0$ and $\delta \mathbf{B}_r / \delta z = \delta \mathbf{B}_z / \delta r$. Further, because the magnitude of $\delta \mathbf{B}_z / \delta r$ is much smaller than $\delta \mathbf{B}_z / \delta z$ and $\delta \mathbf{B}_r / \delta r$, its contribution to the overall magnetic force can be neglected, resulting in:

$$\mathbf{F}_m = \begin{bmatrix} \mathbf{m}_r \dfrac{\delta \mathbf{B}_r}{\delta r} & \mathbf{m}_z \dfrac{\delta \mathbf{B}_z}{\delta z} \end{bmatrix}^T \tag{S14}$$

which is

$$\mathbf{F}_m = [-\mathbf{m}_r \, g_z/2 \quad \mathbf{m}_z g_z]^T \tag{S15}$$

At equilibrium, the vertical forces are balanced, and $\mathbf{F}_z = \mathbf{F}_c + \mathbf{F}_b - \mathbf{F}_g = 0$. This equation can be solved to determine the value of g_z required to keep the microrobot floating, yielding

$$g_0 = \left(\mathbf{F}_g - \mathbf{F}_b\right) / \mathbf{m}_z \tag{S16}$$

To drive the microrobot, we include an additional magnetic force for the propulsion of the robot, $\mathbf{F}_l = \mathbf{m} \cdot \mathbf{G}_l$, where $\mathbf{G}_l = [g_{lr} \quad g_{lz}]^T$. Hence, the required magnetic gradient is

$$\mathbf{G}_m = [g_{lr} \quad g_0 + g_{lz}]^T \tag{S17}$$

Because a vertical force must be exerted on the microrobot to compensate the gravity force, the axis of ***m’*** and the magnetic force does not match the direction of motion (φ) of the microrobot. Knowing that $g_{lz} = G_l \sin\varphi$, $g_{lr} = G_l \cos\varphi$, and $G_l = \sqrt{g_{lz}^2 + g_{lr}^2}$, we defined the following system of equations using Equation S17 and S14

$$\begin{aligned} g_z \sin\psi &= g_0 + G_l \sin\varphi \\ g_z/2 \cos\psi &= G_l \cos\varphi \end{aligned} \tag{S18}$$

where G_l is the magnitude of vector $\boldsymbol{G_l}$. Solving for ψ and g_z we obtain

$$\psi = \arctan\left(\frac{g_0 + g_{lz}}{2 g_{lr}}\right) \tag{S19}$$

$$g_z = \frac{g_0 + g_{lz}}{\sin\psi} \tag{S20}$$

Consequently, we can control the motion and the orientation of a microrobot in the three-dimensional space (x, y, z) using magnetic forces, by controlling the magnitude of the magnetic field gradient produced by the H_z coils, and the direction of the magnetic field, with the following expression

$$\mathbf{B} = \left[B\cos\psi\cos\theta \quad B\cos\psi\sin\theta \quad B\sin\psi \right] \tag{S21}$$

Locomotion experiments herein aimed to demonstrate the different types of automatic locomotion that can be implemented using MECS. Therefore, the above equations were used for a simple implementation of closed-loop controllers. However, for the development of a more precise automatic locomotion control algorithm, additional forces such as wall effects and interactions between the robots must be considered. For instance, the drag force for a spherical particle in a flowing fluid, including wall effects, is given by[6]

$$\mathbf{F}_d = -\frac{\rho_f}{2}\left[\frac{1+\left(\lambda/\lambda_0\right)^{\alpha_0}}{1-\lambda^{\alpha_0}}\left(\boldsymbol{v}-\boldsymbol{v}_f\right)\right] A C_d \frac{\left(\boldsymbol{v}-\boldsymbol{v}_f\right)}{\left\|\boldsymbol{v}-\boldsymbol{v}_f\right\|} \tag{S22}$$

where ρ_f is the fluid density, A is the frontal area of the robot, C_d is the drag coefficient, $\boldsymbol{v}$ is the velocity of the robot, $\mathbf{v}_f$ is the fluid's velocity, $\lambda = 2r/D$ is the robot diameter ratio with D being the vessel (or channel) diameter, and λ_0 and α_0 are the functions of Reynolds number, commonly set to 1.5 and 0.29, respectively.

The magnetic dipolar interactions between two robots α and β can be calculated as[7]

$$\mathbf{F}_{\alpha\beta}^{m} = -\nabla U_{\alpha\beta} = \frac{3\mu_0}{4\pi r_{\alpha\beta}^5}\left[\left(\mathbf{m}_\alpha \cdot \mathbf{r}_{\alpha\beta}\right)\mathbf{m}_\beta + \left(\mathbf{m}_\beta \cdot \mathbf{r}_{\alpha\beta}\right)\mathbf{m}_\alpha + \left(\mathbf{m}_\alpha \cdot \mathbf{m}_\beta\right)\mathbf{r}_{\alpha\beta} - 5\frac{\left(\mathbf{m}_\alpha \cdot \mathbf{r}_{\alpha\beta}\right)\left(\mathbf{m}_\beta \cdot \mathbf{r}_{\alpha\beta}\right)}{r_{\alpha\beta}^2}\mathbf{r}_{\alpha\beta}\right] \tag{S23}$$

where $\mathbf{m}$ is the respective magnetic moment, and $\mathbf{r}_{\alpha\beta}$ is the distance between the robots.

Section 5. Detailed temperature measurements of temperature increase control of magnetic nanoparticles experiments

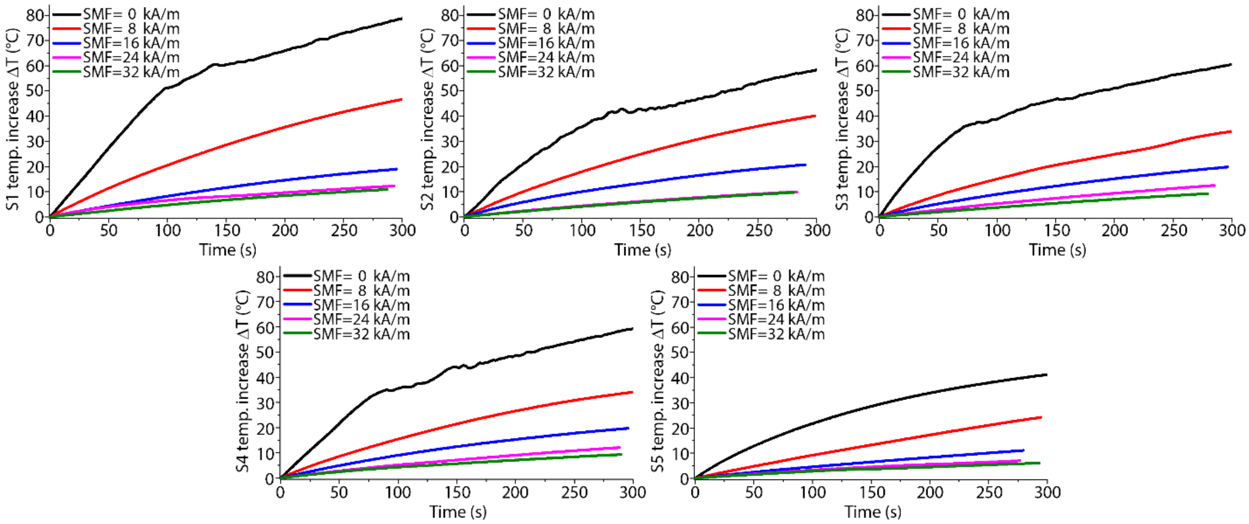

Figure S9. Temperature increase of MF1. Temperature increase for the five samples of MF1 when exposed to different values of SMF.

For analyzing the temperature increase (ΔT) of MF1 when exposed to an AMF of 15.6 kA m^{-1} and SMF values ranging from 0 to 32 kA m^{-1}, we prepared five vials (S1 to S5) with 2 ml of MF1 each. As can be observed in **Figure S9**, the ΔT was similar for S2, S3 and S4, but it varied significantly for S1 and S5. Although we used a tip sonication to disperse the fluid before the experiments, it appears that the MNP were not evenly distributed along the MF1, which explains the variability in the data. However, when we normalized ΔT according to the maximum ΔT of each sample, we observed similar trends. Thus, we normalized the ΔT values and considered the average to create the graph shown in Figure 5D.

Figure S10 shows the temperature measured in °C at each point in the acrylic container that was filled with MF2, from P_1 to P_{13}. As can be observed, the temperature shows a similar decrease in the measured temperature with the increase in the value of the applied SMF. The temperature of the points found at the edge was higher because the magnetic field produced by the induction coil was slightly higher at that location. In addition, the temperature at P4 is significantly lower than the temperature measured at the other points, suggesting that the concentration at that point was lower. However, P4 followed the same reduction in the temperature with the increase in the SMF value.

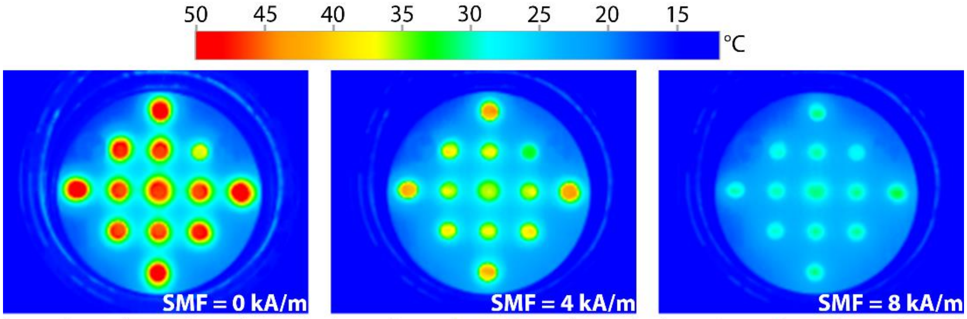

		49.8°		
	47.5°	46.5°	36.5°	
50.1°	46.2°	46.5°	46.4°	50.8°
	46.2°	46°	47.6°	
		48.8°		

		42.1°		
	36.8°	36.2°	31.9°	
41.1°	35.8°	35.5°	35.9°	42.4°
	36.7°	36.1°	37.3°	
		40.3°		

		29.8°		
	28.6°	29.6°	28.0°	
29.0°	29.3°	30.4°	29.8°	30.9°
	28.7°	29.6°	29.6°	
		29.7°		

Figure S10. Temperature increase of MF2. Temperature measured at each point containing MF2 when exposed to an SMF of 0, 4, and 8 kA m^{-1}.

Section S6. Detailed temperature measurements for focused heating of MNP experiments

Figure S11 and **S12** show the temperature measured at each point containing MF2 for the experiments on focused heating using an FFR. Figure S11 shows the temperature when controlling the size of the FFR through the value of the magnetic gradient ∇B_r (**G**r), whereas Figure S12 shows the temperature when the position of the FFR was changed.

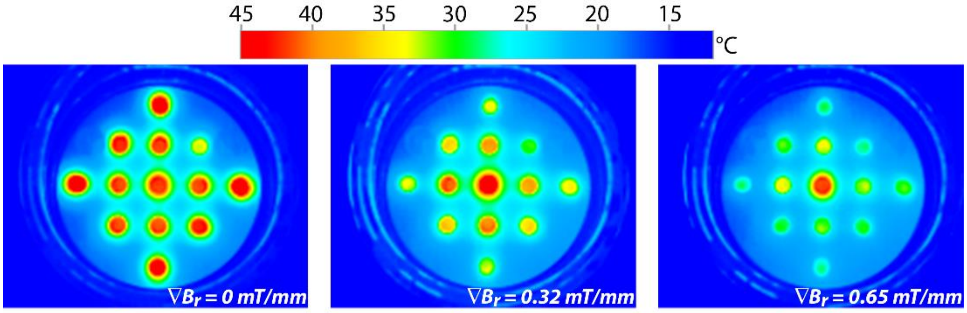

		49.8°		
	47.5°	46.5°	36.5°	
50.1°	46.2°	46.5°	46.4°	50.8°
	46.2°	46°	47.6°	
		48.8°		

		31.9°		
	34.9°	38.1°	29.7°	
31.9°	40.7°	45.6°	37.6°	33.3°
	35.6°	39.7°	34.6°	
		31.7°		

		27.3°		
	29.9°	32.0°	27.1°	
26.8°	33.1°	41.6°	31.4°	29.8°
	28.6°	30.0°	28.2°	
		26.3°		

Figure S11. FFR size control. Temperature measured at each point containing MF2 when exposed to an ∇B_r of 0, 0.32, and 0.65 mT mm^{-1}.

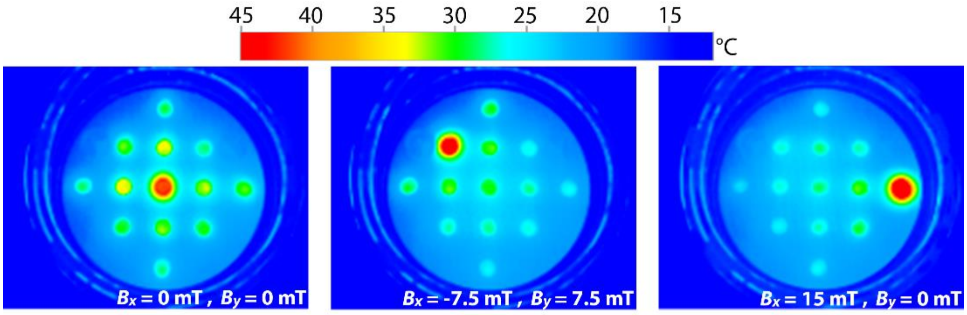

		27.3°		
	29.9°	32.0°	27.1°	
26.8°	33.1°	41.6°	31.4°	29.8°
	28.6°	30.0°	28.2°	
		26.3°		

		28.5°		
	44.7°	30.3°	25.9°	
28.1°	30.5°	29.3°	27.2°	25.4°
	26.8°	27.3°	26.0°	
		24.8°		

		24.7°		
	26.4°	27.5°	26.9°	
23.2°	26.4°	27.9°	30.2°	49.7°
	24.7°	26.4°	28.4°	
		25.0°		

Figure S12. FFR position control. Temperature measured at each point containing MF2 when the FFR is moved from P_7 to P_2 and P_9.

Supporting Videos

Video S1: Magnetic torque and magnetic force locomotion control.

Video S2: Single and collective locomotion of microrobots using trapping point.

Video S3: Temperature control of MF1 using SMF.

Video S4: FFR control-based focused heating of MNP.

Video S5: Selective melting of magnetic jellies using SMF.

Video S6: Hard-magnetic microrobot locomotion and eddy currents heating with suppression of MNP heating using an SMF.

Video S7: Soft-magnetic microrobot locomotion and targeted heating using FFR.

Video S8: Exemplification of targeted therapy in an artificial body fluid.

References

[1] Q. Cao, X. Han, B. Zhang, L. Li, *IEEE Transactions on Applied Superconductivity*, **2012**, 22, 4401504–4401504.

[2] H. Choi, K. Cha, S. Jeong, J.-O. Park, S. Park, *IEEE/ASME Trans. Mechatronics*, **2013**, 18, 1221-1225.

[3] Q. Zhang, S. Song, P. He, H. Li, H.-Y. Mi, W. Wei, Z. Li, X. Xiong, Y. Li, *IEEE Access*, **2020**, 8, 71083–71092.

[4] K. T. Nguyen, M. C. Hoang, G. Go, B. Kang, E. Choi, J.-O. Park, C.-S. Kim, *Control Engineering Practice*, **2020**, 97, 104340.

[5] A. Ramos-Sebastian, S. H. Kim, *IEEE Access*, **2021** , 9, 128755–128764.

[6] L. Arcese, M. Fruchard, A. Ferreira, *IEEE Transactions on Biomedical Engineering*, **2012**, 59, 977-987.

[7] H. Xie, M. Sun, X. Fan, Z. Lin, W. Chen, L. Wang, L. Dong, Q. He, *Science Robotics*, **2019**, 4, aav8006.
